# Fused Tricyclic Guanidine Alkaloids: Insights into Their Structure, Synthesis and Bioactivity

**DOI:** 10.3390/md20090579

**Published:** 2022-09-17

**Authors:** Nur Zahirah Abd Rani, Yean Kee Lee, Sarfraz Ahmad, Ramu Meesala, Iskandar Abdullah

**Affiliations:** Drug Design and Development Research Group, Department of Chemistry, Faculty of Science, University of Malaya, Kuala Lumpur 50603, Malaysia

**Keywords:** marine sponges, tricyclic guanidine, guanidine alkaloids, batzelladines, ptilocaulins, netamines, mirabilins, bioactivities, synthesis

## Abstract

A marine natural product possesses a diverse and unique scaffold that contributes to a vast array of bioactivities. Tricyclic guanidine alkaloids are a type of scaffold found only in marine natural products. These rare skeletons exhibit a wide range of biological applications, but their synthetic approaches are still limited. Various stereochemical assignments of the compounds remain unresolved. Batzelladine and ptilocaulins are an area of high interest in research on tricyclic guanidine alkaloids. In addition, mirabilins and netamines are among the other tricyclic guanidine alkaloids that contain the ptilocaulin skeleton. Due to the different structural configurations of batzelladine and ptilocaulin, these two main skeletons are afforded attention in many reports. These two main skeletons exhibit different kinds of compounds by varying their ester chain and sidechain. The synthetic approaches to tricyclic guanidine alkaloids, especially the batzelladine and ptilocaulin skeletons, are discussed. Moreover, this review compiles the first and latest research on the synthesis of these compounds and their bioactivities, dating from the 1980s to 2022.

## 1. Introduction

The marine natural product has excellent potential as a drug source. Between 1969 and 2018, eight approved drugs of marine natural product origin were reported [1]. These eight drugs have anti-cancer, antiviral, and analgesic properties, and can be used for treating cardiovascular disease. Six of these drugs were approved in the 2000s, indicating the growing interest in marine natural products in the pharmaceutical area. The harsh environment of the deep sea, in terms of its pressure, light source, temperature, and salinity, causes the marine organism to adopt a different biochemical pathway that might result in a novel mode of action in regard to its bioactivity [2]. The only downfall is the difficulty of isolating the compounds from marine organisms, requiring advanced technology or extensive labor, which limits their further study. Spongistatin, for instance, is one of the most active compounds exhibiting antitumor activity (IC_50_: 10^−10^ M for colon cancer, 10^−12^ M for breast cancer cells). Upon the isolation of three tonnes of sponge, only 0.8 mg of Spongistatin was isolated, limiting the further study of its physiological activity [3]. In terms of structure, marine natural products have larger skeletons than terrestrial natural products, and usually, their skeletons are interlinked by an ester bond [4]. Additionally, marine natural products contain more nitrogen and halogen atoms, which suggests that they can be synthesized through more diverse biosynthetic pathways. One type of marine natural product that has garnered a great deal of attention is the marine guanidine alkaloids. Guanidine has high basicity that causes it to bind different functional groups, such as metals, carboxylates, and phosphates, increasing its biological properties [5]. The guanidine alkaloids can be classified into four main chemotypes: bicyclic or monocyclic guanidine, tricyclic guanidine, polycyclic guanidine, and alkaloids that contain more than one guanidine moiety [6]. Among these chemotypes, the synthesis of tricyclic guanidine is less widely reported and is studied, herein, in this review. 

**Figure 1:**
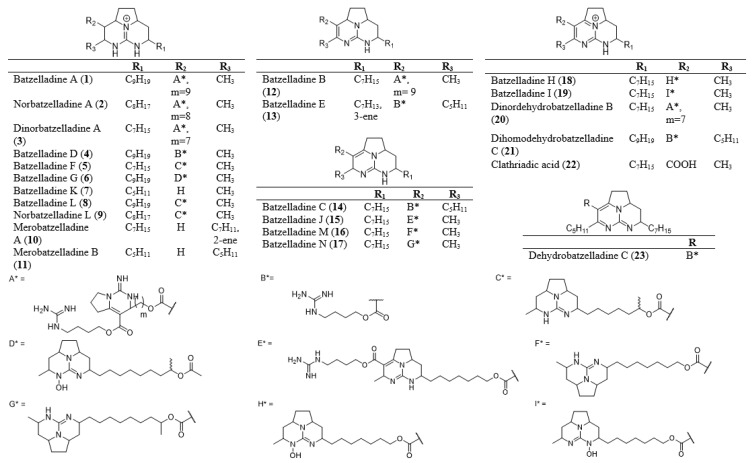
Structures of batzelladines.

**Figure 2:**
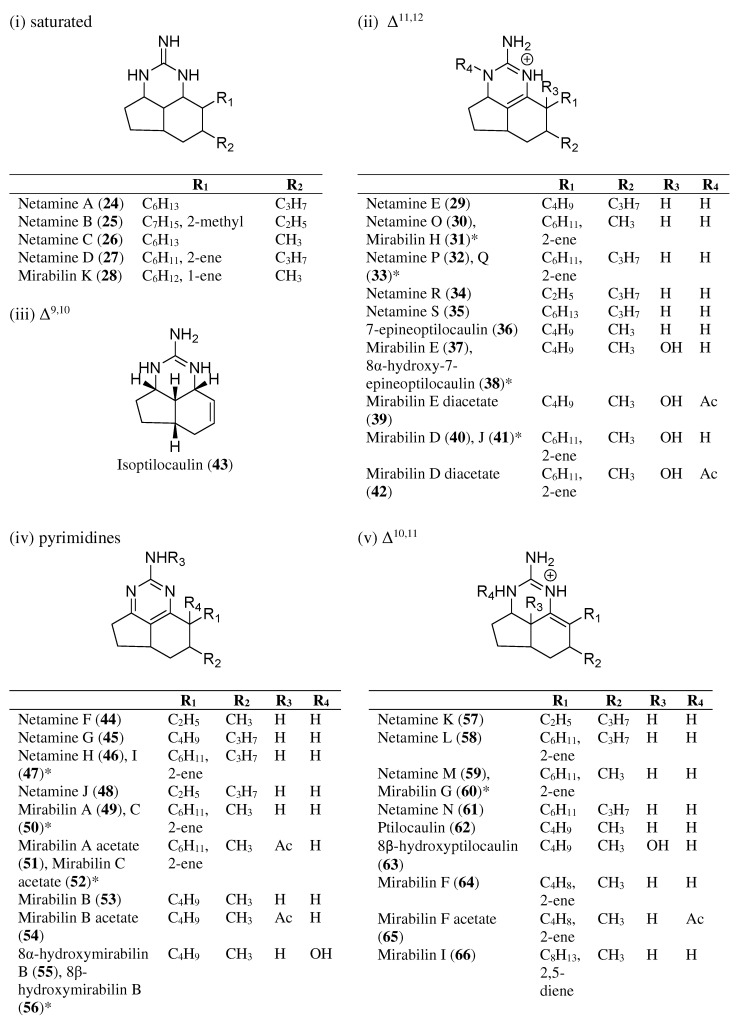
Structures of ptilocaulin, netamine, and mirabilin analogs. * Stereoisomer.

Batzelladines are a type of tricyclic guanidine that are mostly linked through an ester linkage to another guanidine moiety (Figure 1). These guanidine moieties can be 4-guanidino-butyl, bicyclic guanidine, and tricyclic guanidine. The simplest structure of batzelladine contains only one tricyclic guanidine core, such as batzelladine K (**7**) or merobatzelladine B (**11**). These tricyclic guanidine cores also have different degrees of unsaturation that may affect their bioactivities (Table 1). On the other hand, ptilocaulins, mirabilins, and netamines from marine sponges possess a rare heterocycles skeleton, having a tricyclic (5,6,8b)-triazaperhydroacenaphthylene structure without any ester linkage (Figure 2) [7]. In contrast to batzelladines that have an embedded guanidine structure between the tricyclic rings, the ptilocaulins, mirabilins, and netamines have a terminal guanidine skeleton that is positioned on one of the six-membered rings. These skeletons can also be grouped, depending on the degree of unsaturation and double bond positions, i.e., pyrimidines, saturated heterocycles, Δ^9,10^, Δ^10,11,^ and Δ^11,12^ [7]. These alkaloids are reported to have various biological activities, such as antimicrobial [8,9], anti-malarial [10,11,12], antifungal [10], and antiprotozoal properties [10] (Table 2 and Table 3). They are also cytotoxic for specific tumor and leukemia cells [9,13].

In 1995, Patil et al. discovered the first set of compounds from batzelladine skeletons, which were batzelladines A–E (**1, 4, 12**–**14**) from Batzella sp. [14]. Among these compounds, batzelladine A (**1**) and B (**12**) showed significant anti-HIV activity by inhibiting gp120-CD4 binding [14]. These compounds were also the first low-molecular-weight natural products that could inhibit the gp120-CD4 interaction. The presence of three guanidine moieties in batzelladine A (**1**) and B (**12**) might be responsible for their bioactivity. Two years later, Patil et al. isolated new batzelladines from Jamaican Batzella sp. [15]. These batzelladines showed a unique structural feature with two tricyclic guanidines connected through an ester linkage. Batzelladine F (**5**) and G (**6**) and a combination of H (**18**) and I (**19**) exhibited moderate immunosuppression activity by inhibiting p56^lck^-CD4 dissociation. This immunosuppression activity helps to treat autoimmune diseases such as adjuvant-induced arthritis, murine lupus, and allergic encephalomyelitis. Batzelladine J (**15**) was isolated in 2005 from *Monanchora unguifera*, with two repetitive units of tricyclic guanidine with an additional 4-guanidino-butyl ester moiety [16]. This compound exhibited anti-cancer activity against P-388, DU-145, A-549, MEL-28, and HT-29 cells, with IC_50_ values of more than 1 µg/mL. In 2007, batzelladines K–N (**7**–**8**, **16**–**17**) were isolated from the sponge of *M. unguifera* in Jamaica [17]. Together with dehydrobatzelladine C (**23**), these compounds showed significant activity against protozoa, HIV-1, and AIDS opportunistic infectious pathogens (AIDS-OIs), including Mtb and cancer cell lines, with a wide range of bioactivities. It was observed that batzelladine L (**8**) showed potential as a leading drug among the compounds tested. Batzelladine L (**8**) also showed higher activity against *Mycobacterium intracellulare* (IC_50_: 0.25 µg/mL) and comparable activity against leishmania (IC_50_: 1.9 µg/mL) compared to the positive controls, which were ciprofloxacin, pentamidine, and amphotericin B. The compound showed the most potent activity as an inhibitor of *Mycobacterium tuberculosis*, with a MIC value of 1.68 µg/mL. In addition, batzelladine L (**8**) exhibits anti-cancer activity against A549, DU-145, SK-BR3, SK-MEL-28, PANCL, LOVO-DOX, LOVO, HeLa, HT-29, IGROV, and leukemia L-562. In 2020, it was reported that batzelladine D (**4**) and norbatzelladine L (**9**) inhibit the Pdr5p transporter of Saccharomyces cerevisiae in terms of its catalytic and functional activity [18]. This inhibition induces the reversal of fluconazole resistance once the compounds are consumed together with the azole drug, which helps to counter multidrug resistance in fungi. The newest bioactivity of batzelladine was reported in 2022 when 15 batzelladines were screened for their inhibitory activities against the SARS-CoV-2 main protease (M^pro^) through molecular docking studies [19]. Most of the batzelladines tested showed good binding energy, ranging from −7.12 ± 0.60 to −6.22 ± 0.37 kcal/mol, which is similar to that of the tested native ligands O6K (−7.36 ± 0.34 kcal/mol) and N3 (−6.36 ± 0.31 kcal/mol). Among the batzelladines, the most promising compounds in terms of their anti-SARS-CoV-2 activity were batzelladine H (**18**) and batzelladine I (**19**). Based on a structure-activity relationship study, the factors that affect the protein-ligand interaction were identified as the position of the N-OH functionality, the length of the spacer between the two active sides, and the compounds’ degree of unsaturation. The bioactivities of batzelladines are tabulated in Table 1 and Table 3. 

Before the first batzelladines were found, in 1981, Rinehart et al. isolated isoptilocaulin (**43**) and ptilocaulin (**62**) from the Caribbean sponge *Ptilocaulis spiculifer* [27]. The study also reported their antimicrobial and antitumor activities, with more potent activities exhibited by ptilocaulin (**62**) than isoptilocaulin (**43**). It is cytotoxic for L1210 leukemia cells, with an IC_50_ value of 0.39 µg/mL. It showed a low µg/mL MIC activity against *Escherichia coli*, *Streptococcus pyogenes*, *Streptococcus faecalis*, *Streptococcus pneumoniae,* and *Staphylococcus aureus*. Several years later, the isolation of ptilocaulin derivatives from the sponges *Monanchora arbuscula* and *Batzella* sp. was reported [28,29,30]. In 1996, a similar skeleton derived from ptilocaulin was isolated and became known as mirabilin. Mirabilins A–F (**40, 49, 50, 53, 64**) were isolated in 1996, and mirabilin G (**60**) was isolated in 2001, from *Arenochalina mirabilis* and *Clathria* sp., respectively [31,32]. Mirabilin G (**60**) showed antifungal and antimicrobial activities against *Saccharomyces cerevisiae*, *E. coli*, and *Serratia marcescens* [32]. In terms of anti-cancer activity, mirabilin C (**26**) and mirabilins F–J (**31, 41, 60, 64, 66**) showed moderate activity against intestinal (Intestine-407), gastric (AGS), neuroblastoma (SH-SY5Y), and colorectal (HT29) cancer cell lines [33]. The isolation of new ptilocaulin derivatives, netamines A–S (**24**–**27, 29**–**30, 32**–**35, 44**–**48, 57**–**59, 61**), sparked the interest of natural product chemists [34,35,36]. Netamine K **(57)** and netamines O–Q (**30, 32–33**) exhibited significant anti-malarial activity against *Plasmodium falciparum* [35,36]. Netamines also showed anti-cancer activity towards lung (A549), colon (HT29), breast (MDS-MB-231), and KB cell lines [34,35,36]. Under tumor-promoting conditions, netamine M (**59**) and mirabilin G (**60**) exhibited antitumor activity via PDCD4 stabilization, with EC_50_ values of 2.8 µg/mL and 1.8 µg/mL, respectively [37]. These compounds were the first marine natural products reported with this bioactivity. In combatting the SARS-CoV-2 virus, Ramadhan et al. reported that mirabilin G (**60**) (−7.38 kcal/mol) possesses a similar binding activity with the native ligand N3 (−7.30 kcal/mol) to the SARS-CoV-2 main protease (M^pro^) [38]. The stability of the compound and its good binding affinity to the enzyme prove that it could be a potential target for inhibiting the SARS-CoV-2 virus. 

Despite having unusual skeletons and intriguing bioactivities, these alkaloids are only found in marine sponges, limiting their availability for further research. The scope of bioactivity studies is also constrained by the minute quantities of the compounds isolated (<0.1%) and the high consumption of solvents, time, and money during the isolation and purification processes. To further investigate their biological potential, it is critical to understand the synthesis of these tricyclic guanidine alkaloids. This review is designed to help readers to understand the strategies utilized for synthesizing the tricyclic guanidine alkaloids and, hence, to help researchers to develop more economical routes to improve our understanding of this type of skeleton. 

## 2. Methodology

The literature was obtained from Google Scholar, ScienceDirect, SciFinder, and PubMed. The search terms used were “batzelladine”, “ptilocaulin”, “netamine” and “mirabilin”. In total, 155 related articles were obtained after removing duplicate articles. Only articles written in English were included in the study. All of the 155 articles were evaluated, and only 62 articles were included in this study due to their eligibility for the focus of the study. 

## 3. Synthesis

### 3.1. Batzelladine Skeleton

**Figure 3:**
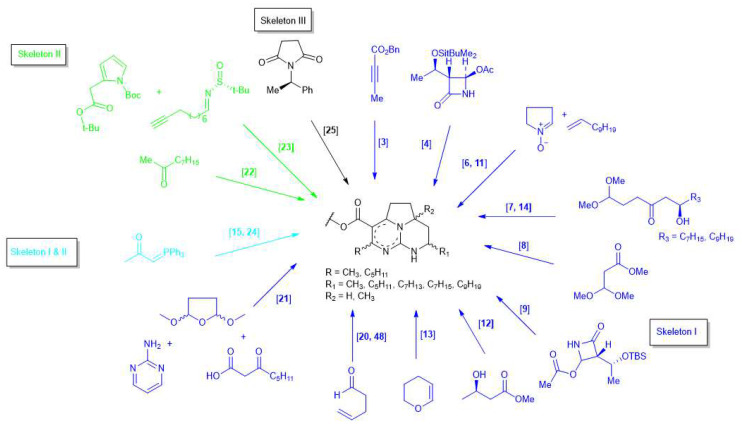
Starting material for synthesizing batzelladine skeleton.

There are five skeletons of batzelladines varying in terms of the degree of unsaturation within their tricyclic guanidine skeletons (Figure 1). Only three of these five skeletons have been synthesized, with most studies focusing on Skeleton I (Figure 3). In most cases, the batzelladine skeleton, comprising a tricyclic guanidine, is linked to another guanidine moiety through an ester linkage. The cyclization of the tricyclic guanidine is commonly conducted through tethered Biginelli condensation, Mitsunobu reaction, or ring-closing iodoamination reaction [3,6,11,12,14,20,22,25]. The coupling of the bicyclic guanidine with the ester linkage must be performed before the cyclization of the bicyclic guanidine into tricyclic guanidine, because the axially orientated ester complicates the coupling reaction [6,43]. The starting material for synthesizing different kinds of batzelladines is visualized in Figure 3.

#### 3.1.1. Batzelladine A

**Figure 4:**
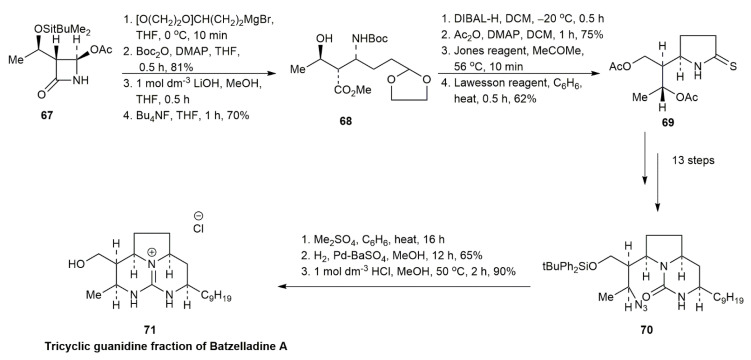
Rama Rao, synthetic approach to the tricyclic guanidine fraction of batzelladine A.

In 1995, Rama Rao et al. reported the first synthesis of the tricyclic guanidine fraction of batzelladine A (Figure 4) [4]. The azetidinone derivative **67** was alkylated through the Grignard reaction. Thiolactam **69** was obtained through the successive reduction of **68**, acetylation, oxidation, and sulfide contraction. Afterwards, 13 steps were conducted to obtain **70**. The tricyclic guanidine fragment of batzelladine A was obtained after the reaction of **70** with dimethyl sulfate, hydrogenation, and desilylation. 

**Figure 5:**
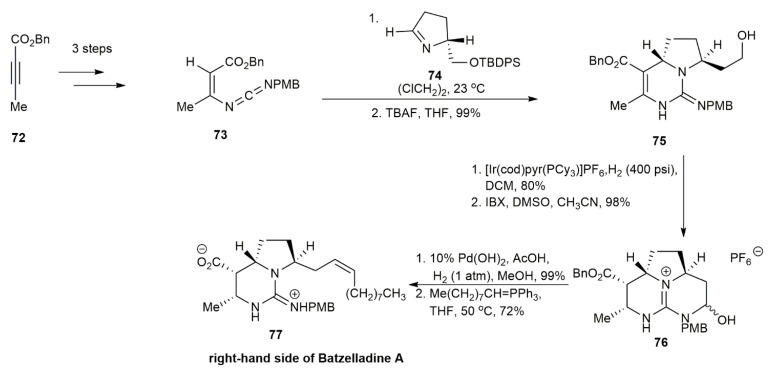
Arnold, synthetic approach to the right-hand side of batzelladine A.

For a long time, the rare annulation of vinyl carbodiimides and imines was only possible using the achiral coupling partner PhCH = NPh [44]. In 2006, Arnold and his co-workers attempted a new strategy for the total synthesis of batzelladine A by performing the annulation using chiral *N*-alkyl imines as the coupling partner [3] (Figure 5). As the starting material, the vinyl carbodiimide was formed from the 1,4-but-2-ynoic acid benzyl ester 72. Then, **72** underwent 1,4 addition and Staudinger–aza-Wittig condensation to form the *E* and *Z* of the vinyl carbodiimides **73**. Additionally, **75** was obtained through the reaction of the previously separated isomer of **73** with the chiral amine 2-(2-*O*-TBDPS-ethyl)-3,4-dihydro-2*H*-pyrrole **74**. The subsequent Ir-catalyzed reduction of **75** followed by IBX oxidation yielded the tricyclic guanidine hemiaminal **76** in a 98% yield. The hydrogenolysis and cis-selective Wittig olefination of **76** afforded the right-hand side of batzelladine A **77** in a 72% yield. 

**Figure 6:**
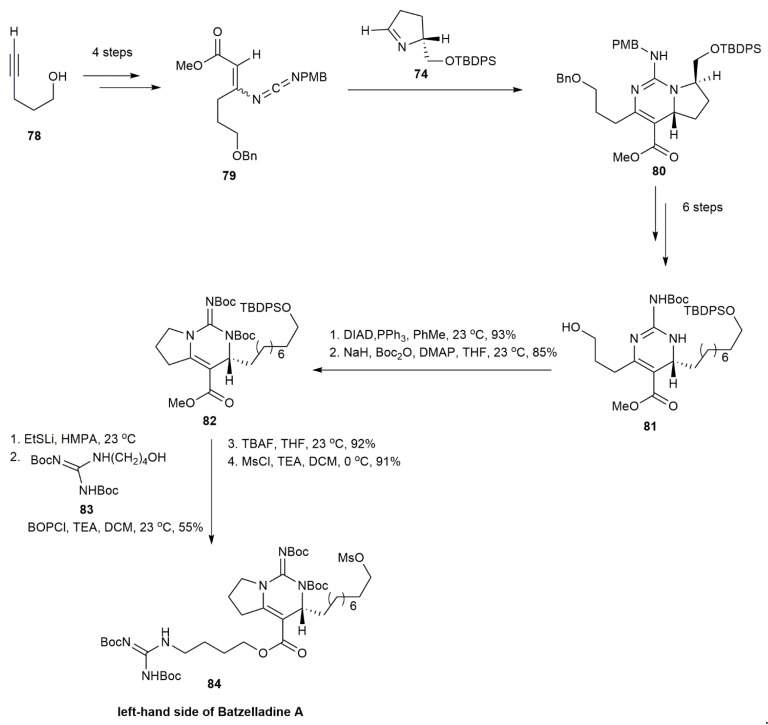
Arnold, synthetic approach to the left-hand side of batzelladine A.

To obtain the left-hand side of the compound, sequential *O*-benzylation and *C*-acylation followed by the 1,4-addition of azide and Staudinger–aza-Wittig condensation of **78** yielded the vinyl carbodiimide **79** in a 63% yield (Figure 6). Racemate **79** reacted with the chiral amine **74** to undergo enantioselective annulation to provide a single diastereomer of dihydropyrimidine of **80** with an 89% yield. Subsequent reactions resulted in a pyrrolidine ring-opening reaction of **80** to form **81**. The ring closure of **81** was accomplished through an intramolecular aza-Mitsunobu reaction to generate the bicyclic guanidine skeleton. The acylation of the compound then afforded the bicyclic vinylogous carbamate **82** in an 85% yield. The vinylogous carbamate **82** was then esterified by **83** and converted into methanesulfonate ester derivative to yield the bicyclic guanidine of batzelladine A **84**. 

**Figure 7:**
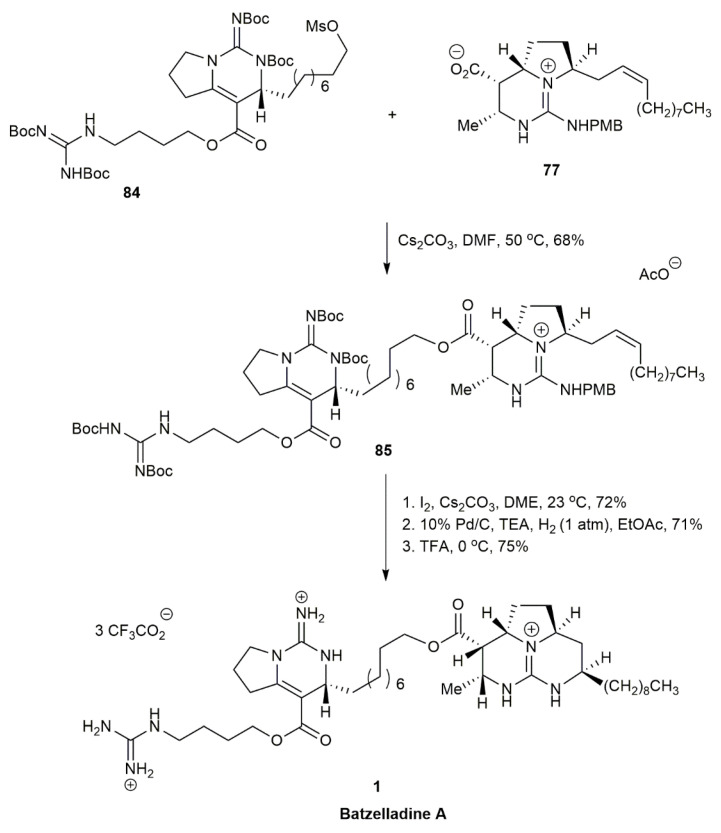
Arnold, synthetic approach to batzelladine A.

Both enantiomers, **77** (right-hand side) and **84** (left-hand side) underwent alkylation to couple both of the compounds by an ester linkage, affording **85** (Figure 7). The intramolecular iodoamination and reductive iodination of **85** produced the cyclization reaction to form the tricyclic guanidine, followed by TFA-mediated deprotection, providing the batzelladine A **1** in a 75% yield. 

#### 3.1.2. Batzelladine D

**Figure 8:**
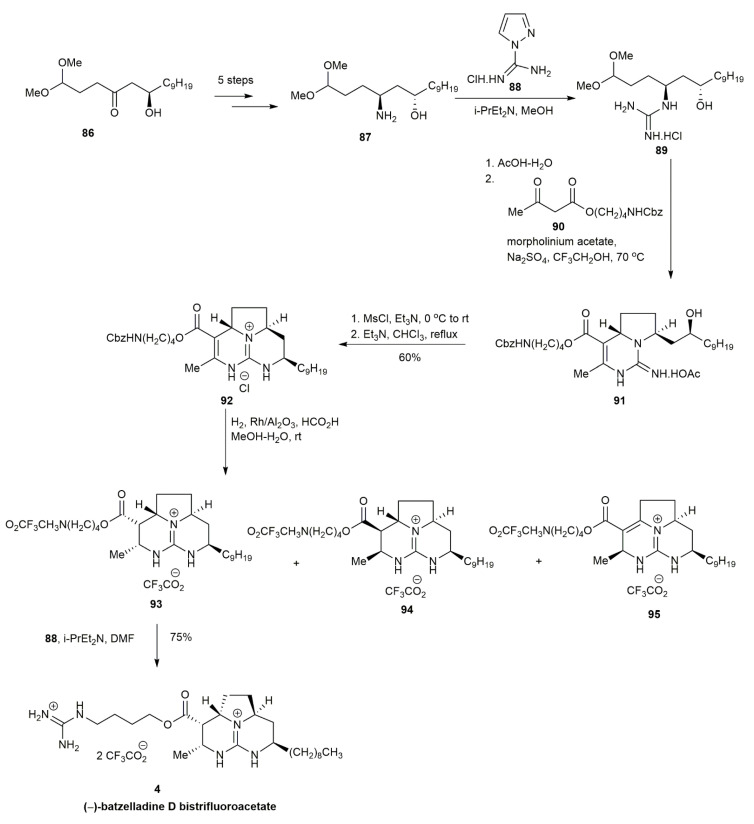
Cohen, approach to (−)-batzelladine D.

In 1999, Cohen and his co-workers reported on the first enantioselective total synthesis of a batzelladine alkaloid through tethered Biginelli condensation, which provided the anti-stereochemistry required to synthesize batzelladine D [7] (Figure 8). The guanylation of **87** with **88** yielded the guanidine **89**. The guanidine was subjected to tethered Biginelli condensation with **90** in the presence of morpholinium acetate and sodium sulfate, affording the guanidium acetate **91** in a 55% yield. The guanidium acetate **91** was mesylated and then cyclized in the presence of triethylamine to provide the tricyclic guanidine **92** in a 60% yield. The subsequent reduction of **92** with Rh/Al_2_O_3_ yielded a mixture of **93**, **94**, and **95**. An optimal product of **93** was obtained when the hydrogenation was conducted at 50 psi. The batzelladine D bistrifluoroacetate **4** was then acquired in a 75% yield upon the guanylation of **88** with **93**. 

**Figure 9:**
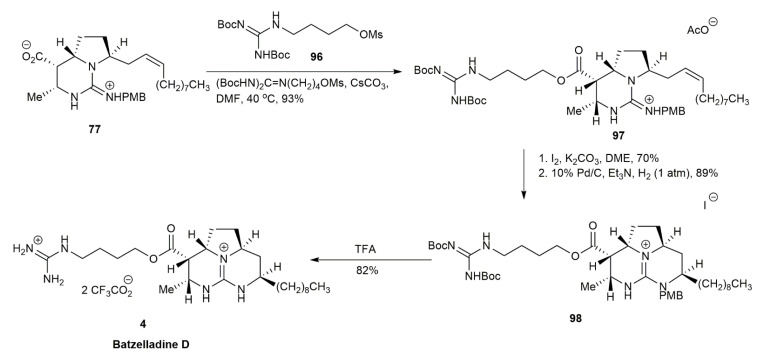
Arnold, synthetic approach to batzelladine D.

As previously reported regarding the synthesis of batzelladine A, intermediate **77** was also used to synthesize batzelladine D [3] (Figure 9). The subsequent *O*-alkylation of **77** with **96** afforded **97** in a 93% yield. The cyclization of bicyclic guanidine was conducted by intramolecular iodoamination and reductive deiodination, followed by deprotection, affording the batzelladine D **4** in an 82% yield. 

**Figure 10:**
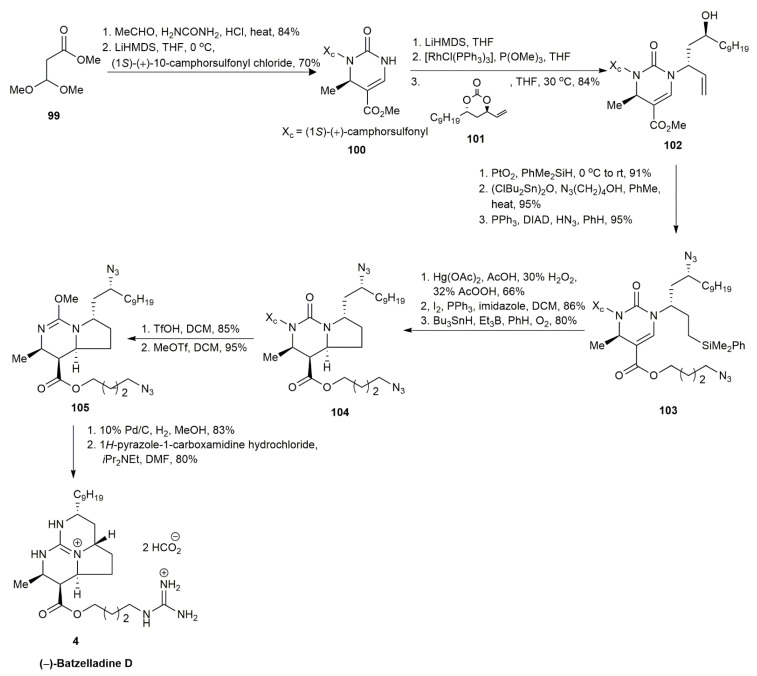
Evans, synthetic approach to (−)-batzelladine D.

In 2007, Evans and co-workers approached the synthesis of (−)-batzelladine D through allylic amination catalyzed by rhodium and free-radical cyclization [8] (Figure 10). The study highlighted the presence of azide in the selective homolytic cleavage of the methyl halide, removing the need for a nitrogen-protecting group. The reaction was initiated by the acid-catalyzed Biginelli condensation of **99** followed by regioselective sulfonylation to afford **100**, which was the fragment required for the allylic amination catalyzed by rhodium. The reaction between the lithium anions of **100** and **101** in the presence of a rhodium catalyst was modified based on Wilkinson’s catalyst, yielding **102** as a mixture of diastereoisomers. The hydrosilylation of **102** followed by transesterification and Mitsunobu inversion afforded **103** in an 82% yield. The diazide **103** was then oxidized using Tamao–Fleming oxidation followed by an Appel reaction to afford alkyl iodide functionality. Subsequently, a radical cyclization with tributyltin hydride and triethylborane produced pyrrolo[1,2-*f*]pyrimidine **104** in an 80% yield. The removal of the camphorsulfonyl group methyl triflate yielded **105** in an 81% yield. The subsequent hydrogenation of **105** followed by cyclization in the presence of an acyclic guanidine moiety afforded (−)-batzelladine D **4** in a good yield. 

**Figure 11:**
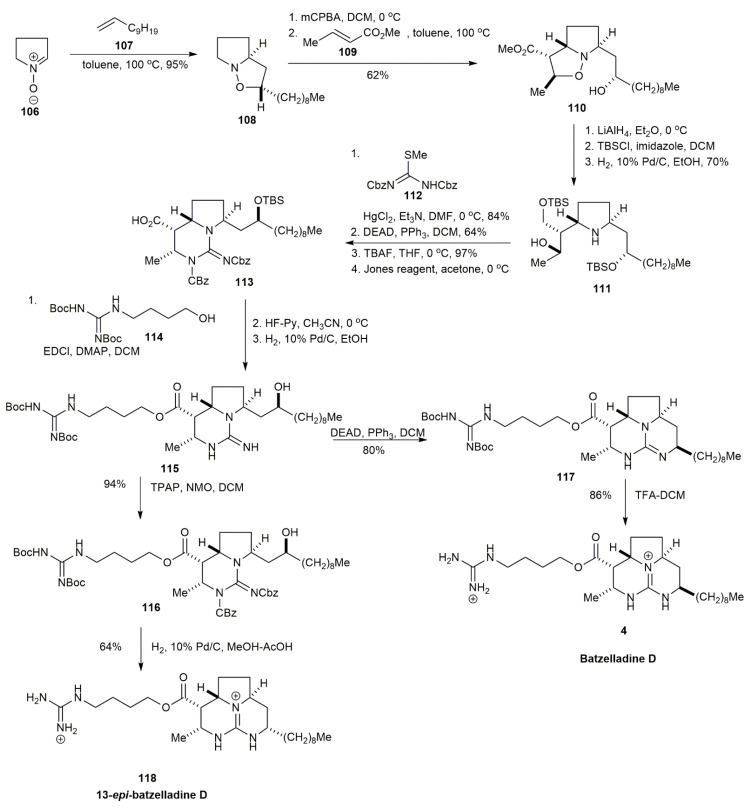
Ishiwata, synthetic approach to batzelladine D and 13-epi-batzelladine D.

In 2002, Ishiwata and co-workers synthesized the absolute stereochemistry of batzelladine D through three main strategies, which were 1,3-dipolar cycloaddition, esterification of the side chain, and tricyclic guanidine formation [6] (Figure 11). The initial strategy was to form the ester linkage between guanidinobutyl alcohol and guanidinecarboxylic acid, but the reaction failed. Similarly, this scenario was also observed by Snider and Chen [43]. It was proposed that the failure was caused by the axially orientated ester or carboxylic acid of the guanidinecarboxylic acid at the C7 position. Hence, the tricyclic guanidine formation was conducted after the coupling of the fragments. The synthesis was initiated by two sequential 1,3-dipolar cycloadditions, alternated with oxidation, yielding isoxazoline **110** in a 62% yield. The sequential reaction of isoxazoline **110** via LiAlH_4_-mediated reduction, followed by hydroxyl protection and then a second reduction with H_2_ on Pd/c, afforded pyrrolidine **111** in a 70% yield. The incorporation of **111** and **112** followed by the Mitsunobu reaction, selective cleavage of silyl ether, and Jones oxidation resulted in bicyclic guanidine **113**. The esterification of **113** with **114**, followed by benzylamino group deprotection, yielded **115**. The Mitsunobu reaction of **115** followed by deprotection afforded batzelladine D **4**, whereas the oxidation of **115** followed by hydrogenation afforded the 13-*epi*-batzelladine D **118**. 

**Figure 12:**
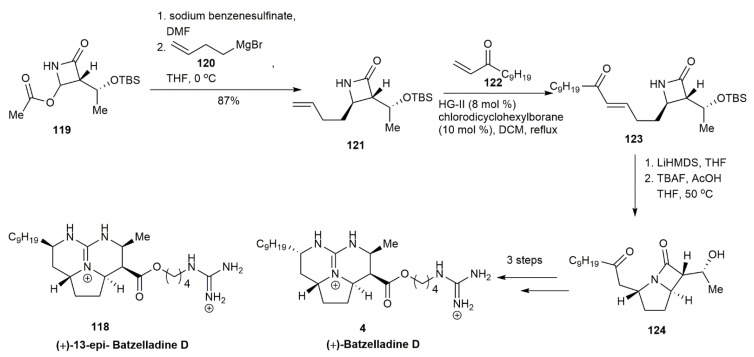
Pierce, synthetic approach to (+)-batzelladine D and (+)-13-epi-batzelladine D.

In 2020, Lin and his co-workers not only managed to synthesize the (±)-batzelladine D, (±)-13-*epi*-batzelladine D and (±)-15-*epi*-batzelladine D, but they also managed to synthesize the single enantiomer of each compound [9] (Figure 12). In this review, only batzelladine D and 13-*epi*-batzelladine D are included. The synthesis of (+)-batzelladine was conducted using *β*-lactam **119,** as it already has the necessary hydroxyethyl side chain as the starting material. The lactam was reacted with sodium benzenesulfinate and Grignard reagent **120** to afford **121** in an 87% yield. The subsequent cross-metathesis of **121** and **122** provided **123,** which underwent aza-Michael addition and TBS deprotection to yield the precursor **124** in an 84% yield. Then, **124** underwent diastereoselective reduction and conversion to yield the (+)-batzelladine D **4** and (+)-13-*epi*-batzelladine D **118**. 

#### 3.1.3. Batzelladine F

**Figure 13:**
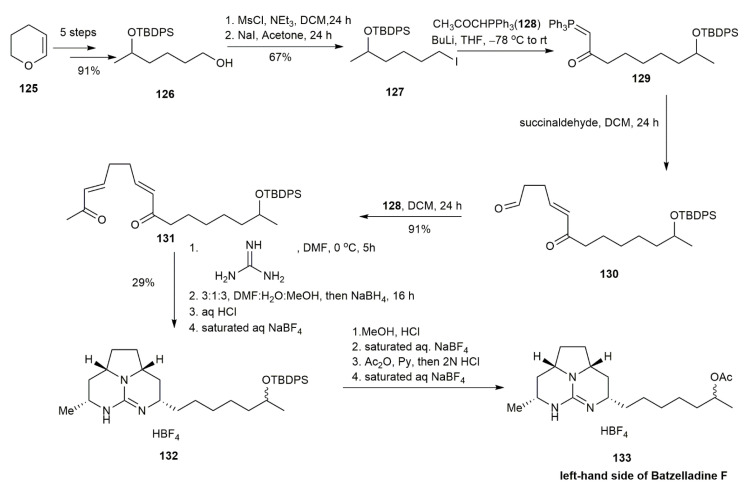
Black, synthetic approach to the left-hand side of batzelladine F.

Batzelladine F is composed of two tricyclic guanidines linked with an ester linkage. It was isolated in 1997, but its ambiguous stereochemical assignment remained a source of debate for several years. In 1998, through biomimetic studies synthesizing ptilomycalin A, Black and co-workers approached the synthesis of the batzelladine skeleton through sequential double Michael additions of guanidine and bis-*α*,*β*-unsaturated ketone [45,46]. This strategy was adapted one year later for the synthesis of the left-hand side of batzelladine F to reveal its absolute configuration [13] (Figure 13). With tetrahydropyran **125** as the starting material, it was converted into **126** in five steps, with a good overall yield of 91%. Sequential mesylation followed by a substitution reaction of **126** with NaI afforded **127** in a 67% yield. The subsequent reaction between **127** and phosphorane **128** generated the ylide **129**, which was followed by the Wittig reaction with succinaldehyde, yielding **130** in a 54% yield. The bis-enone **131** was formed through another Wittig reaction between **130** and phosphorane **128**. The sequential reaction of **131**, involving the addition of guanidine, reduction, and a counter-ion exchange reaction, produced the tricyclic guanidine **132**. The tricyclic guanidine **132** was deprotected and acetylated to form the desired product **133**, the left-hand side of batzelladine F. The study also proposed that the hydrogens bonded to the chiral carbon of the pentacyclic moiety of the tricyclic guanidine **133** were in cis conformation, which readressed the earlier incorrect stereochemical assignment. 

**Figure 14:**
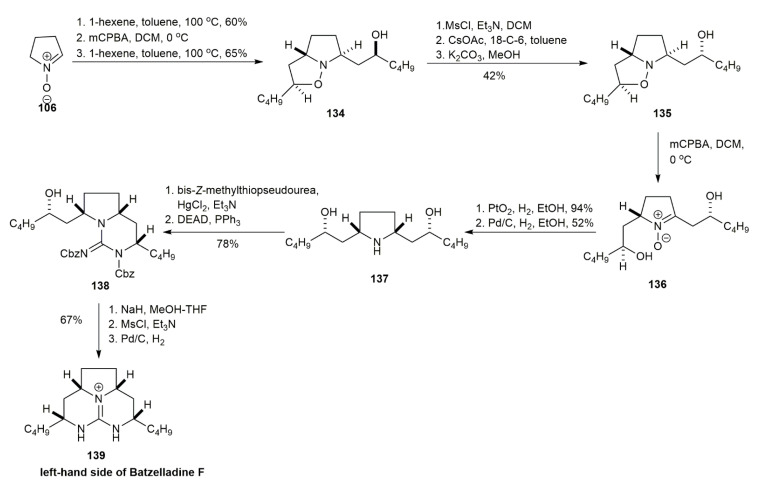
Nagasawa, synthetic approach to the left-hand side of batzelladine F.

The stereochemical assignment of the left-hand side of tricyclic guanidine batzelladine F was further validated by Nagasawa and co-workers via a different synthetic route [11] (Figure 14). The isoxazolidine **134** was obtained from **106** using a similar approach to that for obtaining the isoxazolidine of batzelladine D through 1,3-dipolar cycloaddition, giving a 65% yield [6]. The isoxazolidine **134** was converted to **135** in three steps of the reaction, including mesylation, treatment with cesium acetate, and acetate hydrolysis. The subsequent oxidation of **135** with mCPBA yielded nitrone **136** that, upon reduction, afforded pyrrolidine **137** in a 49% yield. The pyrrolidine **137** was reacted with bis-*Z*-methylthiopseudourea in the presence of mercury (II) chloride and triethylamine to provide the guanylated pyrrolidine. This pyrrolidine was subjected to a Mitsunobu reaction to yield the bicyclic guanidine **138**. The selective deprotection of **138** followed by another mesylation and hydrogenolysis afforded the syn form of tricyclic guanidine **139** in a 67% yield. This study confirmed that the natural batzelladine F possessed syn conformation on the left-hand side of its tricyclic guanidine.

**Figure 15:**
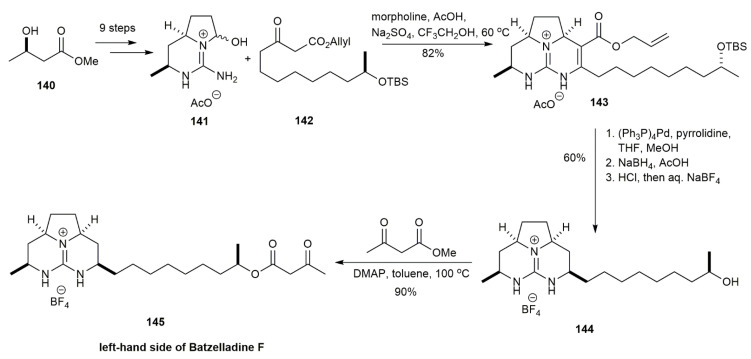
Overman, synthetic approach to the left-hand side of batzelladine F.

In 2006, Overman and Cohen also conducted an extensive study to reveal the absolute configuration of batzelladine F [12] (Figure 15). The synthesis was performed using two different strategies. The first strategy was to couple the alcohol on the left side with the acid on the right side of batzelladine F. This coupling causes epimerization at C19, which prolongs the mechanistic route for successful coupling. Hence, the second strategy was to use a tethered Biginelli reaction to condense the *β*-keto ester of the left-hand side of the batzelladine with the guanidine hemiaminal of its right-hand side. The ring-closing reaction of the right-hand side was conducted, which successfully synthesized the batzelladine F through a shorter route. Upon comparing the products obtained, it was observed that the HPLC of the compound of the natural batzelladine F was different. The mass spectrometry of the authentic batzelladine F suggested that the initial proposal of the compound structure was incorrect. The compound possessed an n-heptyl side chain on its right-hand tricyclic guanidine fragment, instead of the nonyl side chain initially proposed upon isolation. 

The subsequent approach of this study was to synthesize the batzelladine F with the n-heptyl side chain. The synthesis began by converting the hydroxybutyrate **140** into a Weinreb amide, which was subjected to several steps involving a double Mitsunobu reaction, hydrogenation, and then Troc-protected guanidine synthesis, yielding the bicyclic guanidine hemiaminal **141** [12]. The tethered Biginelli reaction between **141** and *β*-keto ester **142** yielded the tricyclic guanidine **143** in an 82% yield. Upon a series of reactions, it afforded the tricyclic guanidine tetrafluoroborate **144** in a 60% yield. The acylation of **144** in the presence of DMAP successfully yielded the left-hand side of batzelladine F **145** in a 90% yield.

**Figure 16:**
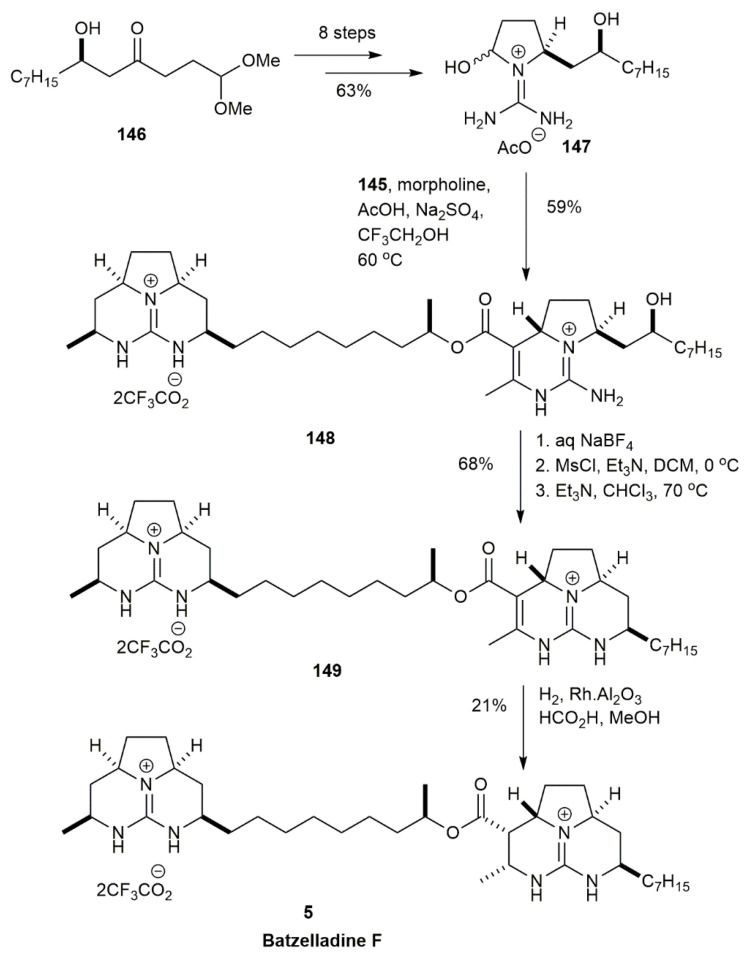
Overman, synthetic approach to batzelladine F.

The right-hand side of batzelladine F was constructed in eight steps of the reaction of the (*R*)-*β*-hydroxy ketone **146** to obtain **147** in a 63% yield [14] (Figure 16). The coupling between the left side **145** with the right-hand side **147** was conducted using a tethered Biginelli condensation reaction, yielding the pentacyclic diguanidine **148** in a 59% yield. The ring-closing of **148** was achieved by exchanging the trifluoroacetate with tetrafluoroborate and converting the compound into a mesylate derivative. The product obtained was hexacycle diguanidine **149** in a 68% yield. The final step was the reduction of the carbon-carbon double bond to yield the batzelladine F **5** in a 21% yield. The stereochemical configuration of the synthesized compound is similar to the stereochemical structure of the authentic batzelladine F.

#### 3.1.4. Merobatzelladine B

**Figure 17:**
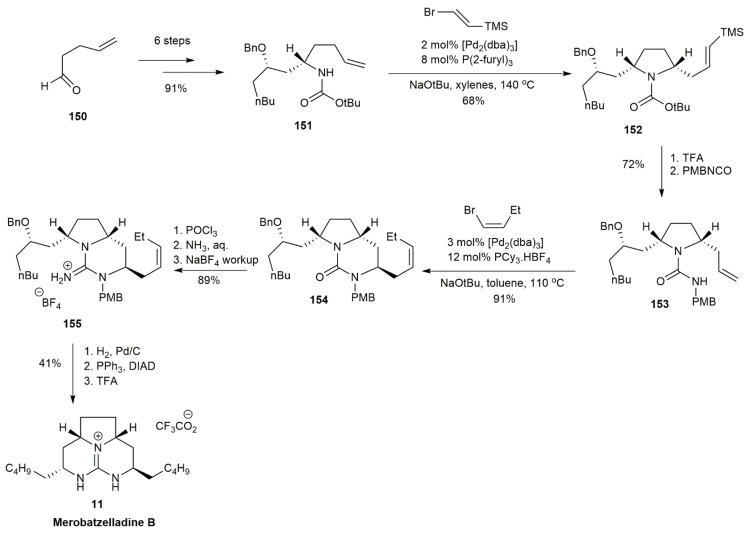
Babij, synthetic approach to (+)-merobatzelladine B.

Merobatzelladine has a unique stereochemical structure compared with batzelladine B. In contrast to batzelladine B, exhibiting an anti-relationship of the C8 alkyl substituents with the C6 hydrogen atom, merobatzelladine has a syn-relationship. In 2012, Babij and Wolfe approached the total synthesis of merobatzelladine B by using a Pd-catalyzed alkene carboamination reaction [20] (Figure 17). The starting material **150** underwent six steps involving the asymmetric Mannich reaction to form compound **151**. The carboamination of **151** with *E*-2-bromovinyltrimethylsilane using the Pd/P(2-furyl)_3_ catalyst afforded the cis-disubstituted pyrrolidine **152** in a 68% yield. The pyrrolidine **152** was obtained with high stereocontrol, with a more than 20:1 diastereomeric ratio. The pyrrolidine **152** was subjected to BOC group cleavage using TFA, followed by photodesilylation and *p*-methoxybenzylisocyanate coupling, yielding the pyrrolidinyl urea of **153** in a 72% yield. The second carboamination of **153** with (*Z*)-1-bromo-1-butene using the Pd/PCy_3_ catalyst generated the bicyclic guanidine **154** in a 91% yield. The functional group interconversion of **154** into guanidium salt **155** was conducted using POCl_3_ followed by an addition reaction with ammonia. The obtained product was washed with aqueous NaBF_4_ to trigger anion exchange to avoid complications during the next step. The subsequent hydrogenation, deprotection, and intramolecular Mitsunobu reaction afforded a ring-closure of **155** into **11** in a 41% yield. The merobatzelladine **11** was obtained in 15 steps, with an overall yield of 6.7%. This study introduced the use of Pd-catalyzed carboamination for constructing 5,6-fused bicyclic urea. 

**Figure 18:**
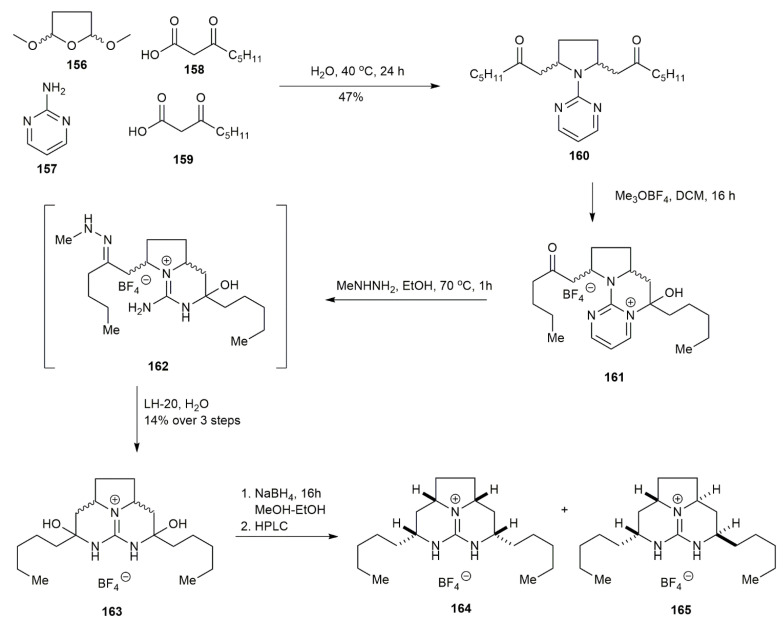
El-Demerdash, synthetic approach to tricyclic guanidine for merobatzelladine B.

The most recent study on tricyclic guanidine was conducted in 2020 by El-Demerdash and his co-workers, using a shorter synthetic route [21] (Figure 18). The study approached the synthesis of a pyrrolidine ring by a multicomponent reaction in one pot, which was initially proposed by Robinson in 1917 for synthesizing tropinone [47]. The multicomponent reaction of this synthesis consists of compounds **156**–**159**, which were dissolved in an aqueous medium for one day to obtain the pyrrolidine **160** in a 47% yield. Under an acidic environment, the heminal **161** was obtained as the result of a nucleophilic attack of the 2-aminopyrimidine on the ketone. The deprotection of the surrogate guanidine was conducted using methyl hydrazine, affording methyl hydrazone **162**. The purification using LH-20 eventually yielded **163** in a 14% yield. The product was then reduced to form **164** and **165.**

#### 3.1.5. 9-*Epi*-batzelladine K

**Figure 19:**
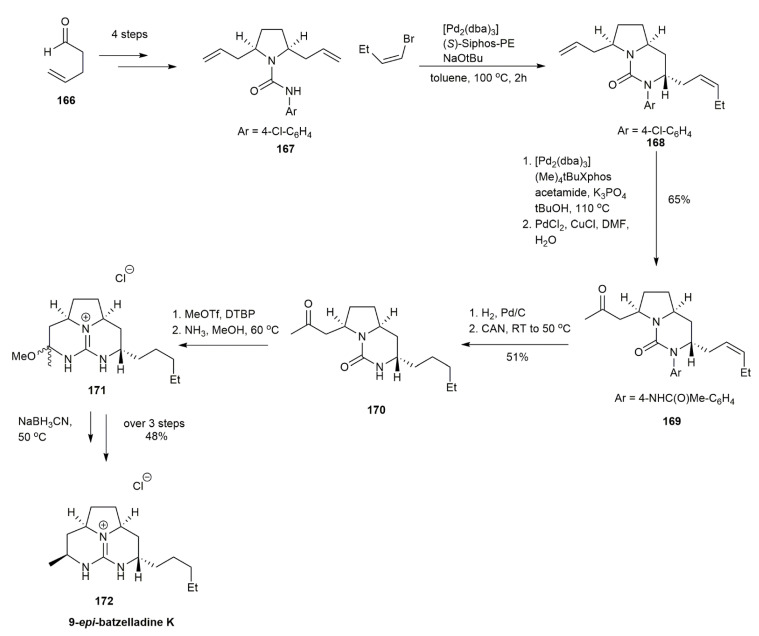
Babij, synthetic approach to 9-epi-batzelladine K.

A previous study by Babij and Wolfe reported the use of a Pd-catalyzed carboamination reaction for constructing bicyclic urea derivatives [48] (Figure 19). The reaction produced a good yield but involved many steps. Hence, they worked on another route via a Pd-catalyzed desymmetrization reaction, which provides the benefit of introducing a different substituent to form highly substituted urea derivatives. They observed that different *N*-aryl substituents affected the asymmetric induction, and the *p*-chlorophenyl substituent afforded the best yield and stereoselectivity. The Pd-catalyzed asymmetric desymmetrization of **167** with 1-bromo-but-1-ene produced **168**, with an exceptional 20:1 diastereomeric ratio. The subsequent cleavage of the *N*-*p*-chlorophenyl group of **168** was performed via Pd-catalyzed *N*-arylation with acetamide followed by Wacker oxidation to avoid base-mediated epimerization, acting against **168**. The Wacker oxidation of **168** produced **169** in a 65% yield. The subsequent hydrogenation and deprotection afforded urea **170** in a 51% yield. The *O*-methylation reaction of **170** and treatment with ammonia afforded the guanidine aminal **171**, which was subjected to reduction and purification, yielding the 9-*epi*-batzelladine K **172** in a 48% yield.

#### 3.1.6. Batzelladine K

**Figure 20:**
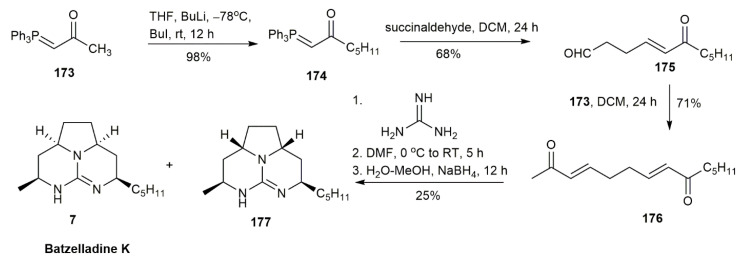
Ahmed, synthetic approach to batzelladine K.

In 2010, Ahmed and his co-workers synthesized batzelladine K in only four steps [15] (Figure 20). The initial idea, contributed by Yu et al., proposed that the addition of guanidine to *α,β*-unsaturated ketone produces a tricyclic guanidium core [40]. The phosphorane **173** was alkylated with 1-iodobutane using butyllithium as the base, affording phosphorane **174**. The reaction was followed by a Wittig reaction between **174** and excess succinaldehyde, yielding the *E*-isomer of **175** in a 68% yield. The ketone **175** was then reacted with phosphorane **173** to form the *α,β*-unsaturated ketone **176**, which upon condensation with guanidine afforded the desired batzelladine K **7** in 25% yield following Michael addition and reduction. The overall product was yielded at 12%.

#### 3.1.7. Batzelladine E

**Figure 21:**
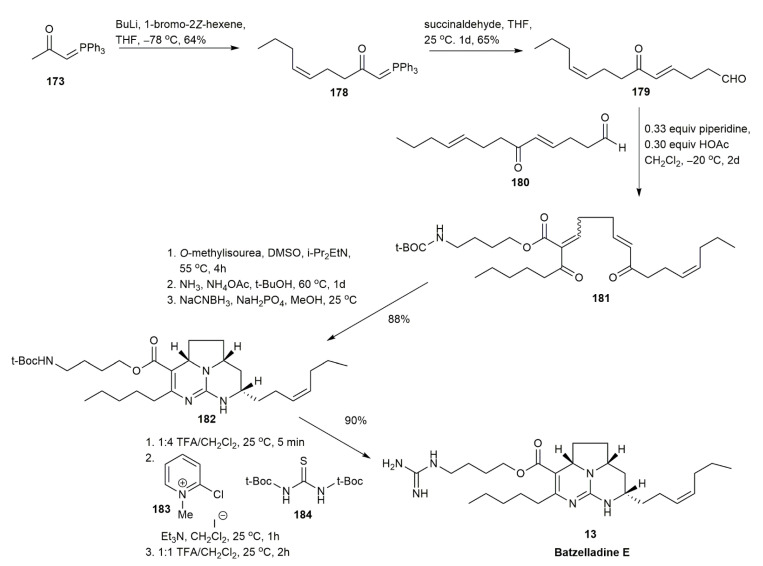
Snider, synthetic approach to batzelladine E.

The first synthesis of the *E* and *Z* isomers of batzelladine E was reported by Snider and Chen in 1998 [24] (Figure 21). Initially, the synthetic approach targeted the *E* isomer, and it was reported that the *E* isomer configuration could be adopted from the naturally isolated batzelladine E. However, upon spectrum comparison, the authors found that the actual configuration of batzelladine E was in the *Z* form. Batzelladine E was synthesized using a similar mechanism as that used for synthesizing a pentacyclic portion of ptilomycalin A [49]. The authors’ earlier studies revealed that the introduction of the ester group could not be achieved after the construction of a polycyclic skeleton [43]. For this reason, the guanidino butyl ester was introduced in the earlier steps of the synthetic route. The deprotonation and alkylation of phosphorane **173** yielded **178** in a 64% yield. Afterwards, **178** underwent two different types of condensation. The first condensation of **178** and succinaldehyde afforded **179** in a 65% yield. Subsequently, the Knoevenagel condensation of **179** and **180** was carried out. Initially, piperidinium acetate was used as the catalyst in the next synthetic strategy, but it was revealed that the *E* isomer was formed. Hence, the catalyst system was changed to 0.33 equivalent of piperidine in the presence of 0.30 equivalent of acetic acid to promote the *Z* isomer formation of the adduct **181**. The subsequent treatment of **181** with *o*-methylisourea followed by ammonolysis and reduction yielded **182** in an 88% yield. The addition of guanidine in the presence of 2-chloro-*N*-methylpyridinium iodide **183** and *N*,*N*’-di-(t-butoxycarbonyl)thiourea **184** generated the batzelladine E **13** in a 90% yield. 

#### 3.1.8. Batzelladine B

**Figure 22:**
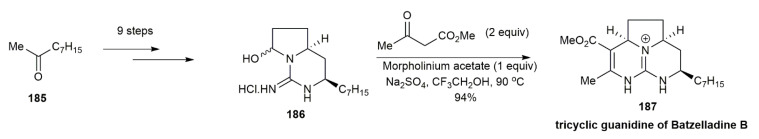
Franklin, synthetic approach to the tricyclic portion of batzelladine B.

In 1999, Franklin and his co-workers applied the tethered Biginelli reaction to construct the tricyclic guanidine skeleton of batzelladine B [22] (Figure 22). The starting material **185** underwent various reactions, including the conversion into Weinreb amide, the addition of guanidine through Mitsunobu displacement, and the reduction of the diazide, producing a diamine. The condensation of the diamine followed by deprotection afforded **186** from **185** in a 32% yield. This intermediate **186** was used for the tethered Biginelli reaction with methyl acetoacetate in the presence of morphonilium acetate and sodium sulfate. The product obtained was the tricyclic guanidine of batzelladine B **187** in a 10:1 isomer ratio, producing an 82% yield upon purification. Although this was the first report on the enantioselective synthesis of the batzelladine B fragment, the tethered Biginelli reaction resulted in low stereoselectivity, and there is still room for improvement. 

**Figure 23:**
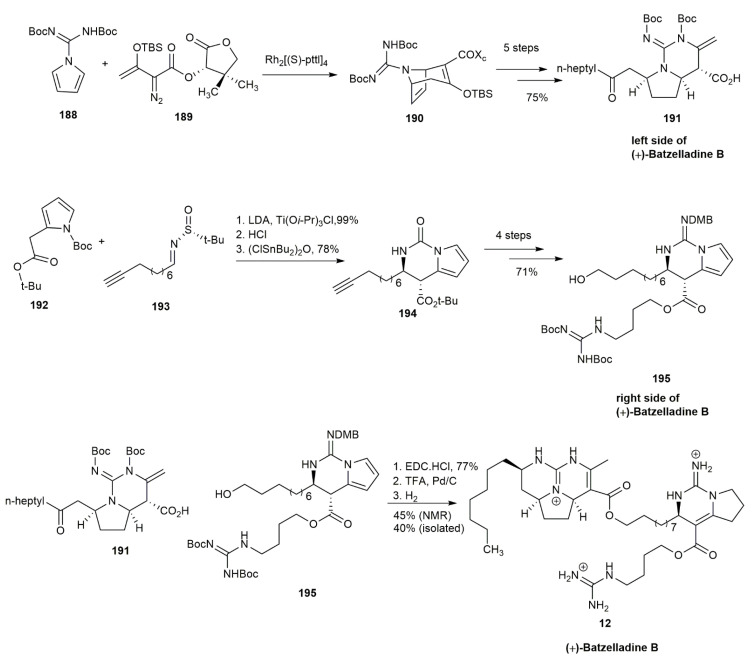
Parr, synthetic approach to (+)-batzelladine B.

In 2015, Parr and his co-workers hypothesized that batzelladine B could be synthesized from pyrrole as a starting material for both sides of the compound [23] (Figure 23). The pyrrole **188** underwent formal cycloaddition with (*S*)-pantolactonyl-α-diazo ester **189** in the presence of dirhodium (II) tetrakis[*N*-phthaloyl-(*S*)-*tert*-leucinate (Rh_2_[(*S*)-pttl]_4_) as the catalyst. The product, **190**, underwent several more reaction steps, including the introduction of TMS-EBX, lithium benzyl octanoate addition, and saponification to yield the left-hand side of (+)-batzelladine B **191**. For the right-hand side of the compound, pyrrole **192** was treated with sulfinimine **193**, followed by the cleavage of the tert-butanesulfinyl substituent and cyclization with bis(chlorodibutyltin)oxide, to afford the urea **194** in a 78% yield. Further *O*-ethylation with 2,4-(dimethoxy)benzylamine hydrogen chloride and the cleavage of the ester afforded a carboxylic acid adduct. This carboxylic acid was then reacted with alcohol followed by anti-Markovnikov reductive hydration and the addition of *p*-toluene sulfonic acid, yielding the right-hand side of (+)-batzelladine B **195**. The addition of *p*-toluene sulfonic acid is crucial for protonating the guanidine. In the next step, EDC·HCl was used to couple both fragments of **191** and **195**. The coupled product was subjected to carbamate cleavage cum cyclodehydration and alkene isomerization upon adding Pd/C. The final step involved regio- and stereoselective reduction, semihydrogenation-isomerization, and DMB cleavage by H_2_. The product, batzelladine B (**12**), was obtained in a 40% isolated yield and a 45% yield based on the NMR spectrum.

#### 3.1.9. Batzelladine C Methyl Ester

**Figure 24:**
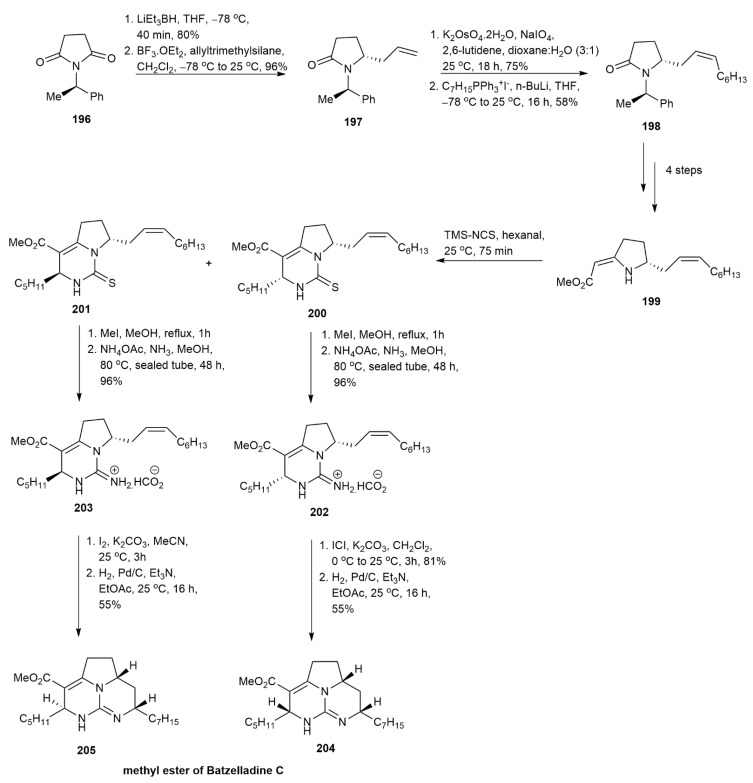
Butters, synthetic approach to batzelladine C methyl ester.

Batzelladine C was isolated in 1995, without the further assignment of its stereochemistry [1]. In 2009, Butters and his co-workers synthesized the methyl ester of batzelladine C by adapting the three-component coupling reaction and iodocyclization that had been developed previously [25,50,51] (Figure 24). These strategies were also used for developing the bicyclic portion of batzelladine A and the tricyclic guanidine of batzelladine D [51,52]. The synthesis route constituted a three-component coupling (aldehyde, alkylidenepyrrolidine, and isothiocyanate), followed by an iodocyclization reaction. The reduction and allylation of **196** yielded **197**, with a good diastereoisomers ratio of 4.4:1. The subsequent cleavage of the terminal double bond followed by a cis-selective Wittig reaction afforded **198** in a 58% yield. The alkylidenepyrrolidine **199** was obtained in several reaction steps, including deprotection, thionation, and Eschenmoser sulfide contraction. The three-component coupling of **199** with hexanal and isothiocyanate afforded a separable mixture of **200** and **201**. The further reaction of **200** and **201** yielded the bicyclic guanidines **202** and **203** in a 96% yield, respectively. The final stage of the reaction was iodocyclization using iodine and potassium carbonate. It was observed that the reaction was successfully applied to **203**, forming the tricyclic guanidine **205**, but this was not the case for the bicyclic guanidine **202**. The tricyclic guanidine **204** was obtained after changing the reagent to iodine monochloride in dichloromethane rather than iodine in acetonitrile. The underlying reason for this difference is unknown, but the modeling studies demonstrated a significant conformational difference between **202** and **203** that might be responsible for this difference. The overall yields of methyl ester batzelladine C **204** and **205** were 1.6% and 4.3%, respectively. Upon the comparison of the spectroscopic data with the authentic batzelladine C, the batzelladine C was observed to have similar stereochemistry to compound **204**.

### 3.2. Ptilocaulin and Its Derivatives Skeleton

**Figure 25:**
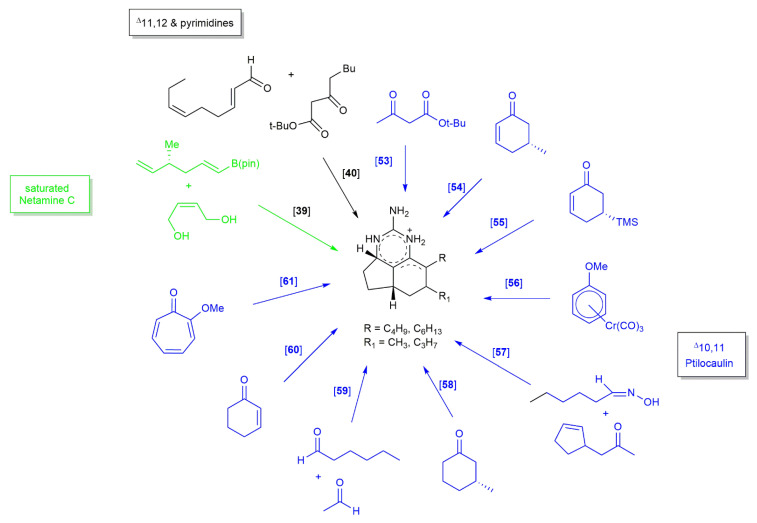
Starting materials for synthesizing ptilocaulin, netamines, and mirabilins skeleton.

Ptilocaulin, netamines, and mirabilins can be classified into five groups based on the degree of unsaturation of their tricyclic skeletons (Figure 2). Until now, only four groups have been successfully synthesized and reported. The skeleton of isoptilocaulin, bearing an unsaturation bond between C9 and C10, has yet to be explored. Most of the synthetic studies have been conducted using skeletons with unsaturation between C10 and C11, specifically targeting only the ptilocaulin structure. Over the years, the tricyclic guanidine of ptilocaulin was synthesized using Snider ketone **209** as its intermediate. Various starting materials and synthetic strategies have been used to form Snider ketone (Figure 25). Most previous studies performed aldol condensation followed by conjugate addition and cyclization to obtain the Snider ketone [40,53,54,55,56]. Before the synthesis of Snider ketones, some studies focused on intramolecular nitrone cyclization to obtain an isoxazolidine, and some studies employed intramolecular nitrogen oxide olefin cyclization to obtain an isoxazoline [57,58]. Various catalysts, such as rhodium and ruthenium complexes, have been used to aid in the reaction [39,59,60]. Subsequently, ptilocaulin was obtained via the addition of guanidine to Snider ketone. Different types of alkyl chains and degrees of saturation of the tricyclic guanidine yielded the netamine and mirabilin derivatives. The alteration of the reaction temperature with subsequent oxidation resulted in different types of tricyclic guanidines. Recently, a study was conducted on the synthesis of a netamine C-bearing saturated tricyclic guanidine skeleton without the performance of Snider ketone synthesis [39]. This study utilized copper-hydride-catalyzed allenylboronate in the reaction. Details of this reaction are discussed in the following section.

#### 3.2.1. Ptilocaulin

**Figure 26:**
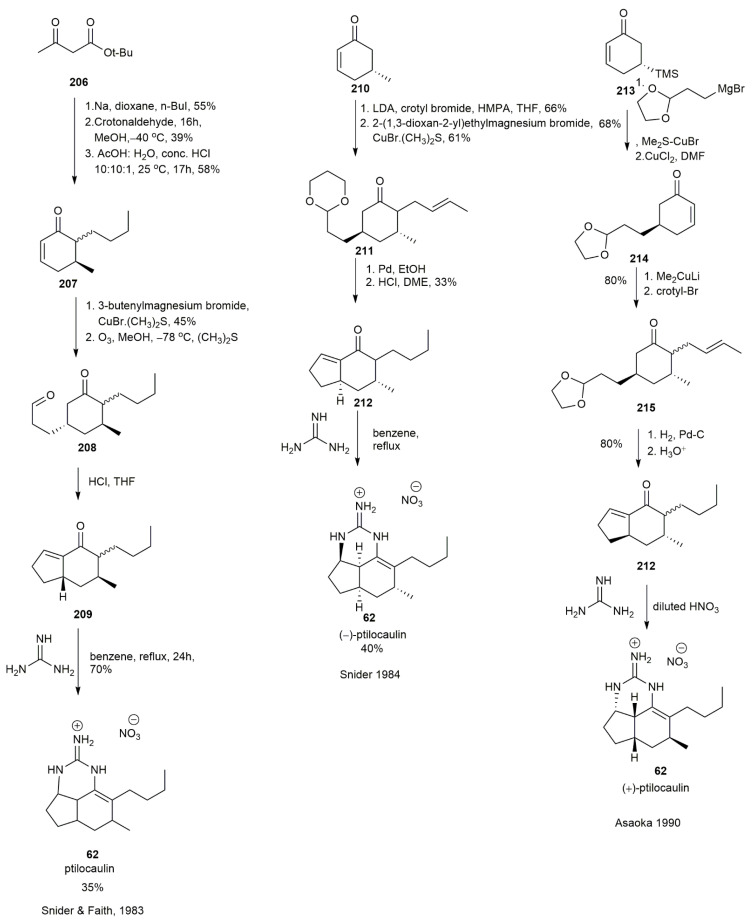
Synthetic approach to ptilocaulin.

The first synthesis of (±)-ptilocaulin **62** was initiated by the Snider group in 1983, two years after the tricyclic guanidine ptilocaulin **62** was isolated by Rinehart et al. [53] (Figure 26). The group synthesized (±)-ptilocaulin **62** in a 35% yield by adding guanidine to a polyketonide chain. The acidic hydrogen of **206** was deprotonated using sodium and displaced by a butyl group. This compound was then subjected to conjugate addition with crotonaldehyde and, upon cyclization and decarboxylation, afforded compound **207**. The subsequent 1,4 addition with 3-butenylmagnesium bromide added another 3-butenyl group to the cyclohexanone and yielded **208** in a 45% yield with a 1.7:1 trans:cis stereoisomer ratio. The functional group transformation of the alkene into a carbonyl was conducted via ozonolysis, followed by intramolecular cyclization to form enone **209** in a chromatographically separable mixture of 1:1. The cis conformation of **209** was refluxed with guanidine in benzene for 24 h in an azeotropic environment to afford the pure ptilocaulin nitrate **62** after elution in silica. The next year, Snider and his co-worker used the same strategy to synthesize the (−)-ptilocaulin [54]. The LDA initiated the alkylation of **210** via enolate chemistry with crotyl bromide and HMPA, followed by the 1,4-addition of **210** using a Grignard reagent, yielding **211** in a 61% yield. The hydrogenation and acid hydrolysis of **211** formed the bicyclic enone **212** in a 33% yield and was converted into ptilocaulin **62**. In 1990, Asaoka and his co-workers synthesized (+)-ptilocaulin **62** using the same strategy employed by Snider and Faith [55]. The reaction began with the conjugate addition of **213** using a Grignard reagent of 3-bromo-propanal ethylene acetal. The subsequent trimethylsilyl group elimination yielded compound **214** in a 68% yield. Another 1,4-addition was conducted using dimethylcuprate with crotyl bromide, affording **215** in an 80% yield. The bicyclic enone **212** was formed from the hydrogenation of **215,** followed by acidic hydrolysis to initiate intramolecular cyclization. The bicyclic enone **212** was then subjected, in the usual manner, to reflux with guanidine followed by treatment with diluted HNO_3_ to afford (+)-ptilocaulin **62** and its C-3a epimer in a 1:1 ratio. 

**Figure 27:**
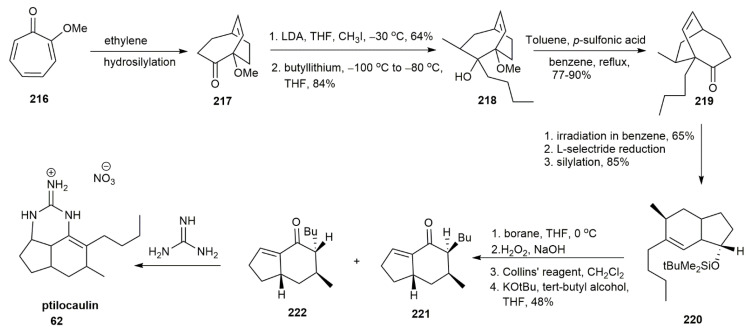
Uyehara’s synthetic approach to (±)-ptilocaulin.

In 1986, Uyehara’s group synthesized (±)-ptilocaulin using another strategy incorporating Diels–Alder and photochemical 1,3-acyl migration reactions [61] (Figure 27). In the synthesis of the 5,6-fused-ring of the Snider ketones **221** and **222**, the tropolone **216** underwent a Diels–Alder reaction with ethylene followed by hydrosilylation, forming the bridgehead methoxy ketone **217.** The alkylation and 1,2-addition of the ketone at a low temperature afforded **218** which, upon the treatment with *p*-sulfonic acid under reflux conditions, afforded a pinacol-type rearrangement, yielding **219** in a 77–90% yield. The irradiation of compound **219** using a 100 W high-pressure mercury lamp triggered the 1,3-acyl migration of the compound. Subsequently, the compound underwent L-selectride reduction and silylation to afford compound **220.** The ptilocaulin intermediates of **221** and **222** were successfully obtained after several reactions in a 1:1 ratio, with a 48% yield. This intermediate was then converted into ptilocaulin **62** by the addition of guanidine. 

**Figure 28:**
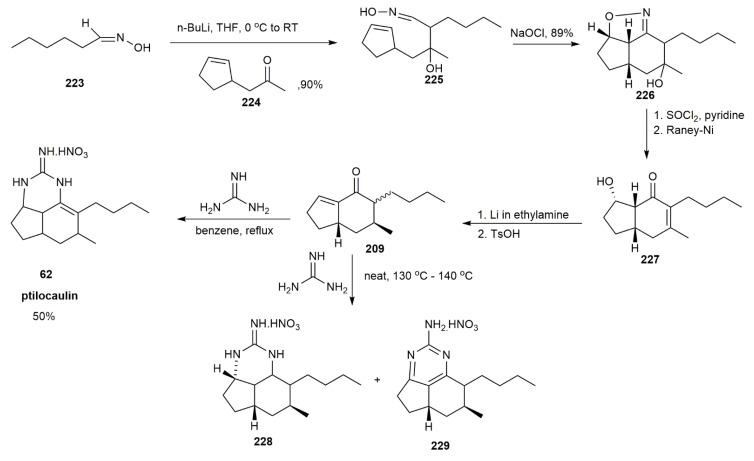
Murthy, synthetic approach to (±)-ptilocaulin.

The intramolecular nitrile oxide olefin cycloaddition (INOC) reaction was also utilized for the formation of ptilocaulin [57] (Figure 28). The aldol condensation between hexanal oxime dianion **223** and ketone **224** yielded a *β*-hydroxy aldoxime **225** in a 90% yield. The isoxazoline **226** was obtained by further reaction of **225** with NaOCl. The isoxazoline **226** was obtained as a combination of four diastereomers that, upon dehydration, afforded unsaturated isoxazoline. Raney-Ni reduction further reduced this isoxazoline to form a single isomer of unsaturated ketol **227** in a 60% yield. Reducing **227** by Li-EtNH_2_ afforded a 7-*β*-methyl ketol, and further dehydration yielded ketone **209**. The subsequent reaction step was the addition of guanidine under reflux conditions to afford ptilocaulin **62**. Interestingly, upon increasing the temperature from 130 ℃ to 140 ℃, a disproportionate product was obtained, with saturated **228** and aromatized cyclic guanidine skeleton **229**. These findings were significant for understanding the formation of ptilocaulin derivatives, such as netamines and mirabilins.

**Figure 29:**
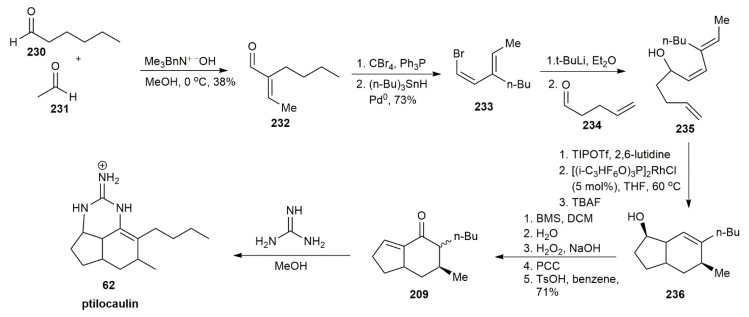
Shen, synthetic approach to (±)-ptilocaulin.

In 2010, Shen and Livinghouse managed to synthesize the (±)-ptilocaulin using an intramolecular [4+2] cycloaddition reaction that was catalyzed by Rh(I) complex [59] (Figure 29). Cross-aldol condensation initiated the reaction between a hexanal **230** and an acetaldehyde **231**. The (*E*)-2-ethylidenehexanal **232** was obtained in a 38% yield. A further reaction with sequential debromoalkylidenation and stereoselective reduction using palladium catalyst yielded (*Z*,*E*)-bromodiene **233** in a 73% yield. The addition of **234** to **233** afforded the alcohol **235**. The protection of alcohol **235** using TIPOtf, followed by intramolecular cycloaddition using the Rh(I) catalyst, yielded the hexahydroindene **236**. The sequential hydroboration-oxidation of **236**, followed by another oxidation using PCC and dehydration, afforded the ketone **209**. Finally, ptilocaulin **62** was obtained after the addition of guanidine to **209**. 

**Figure 30:**
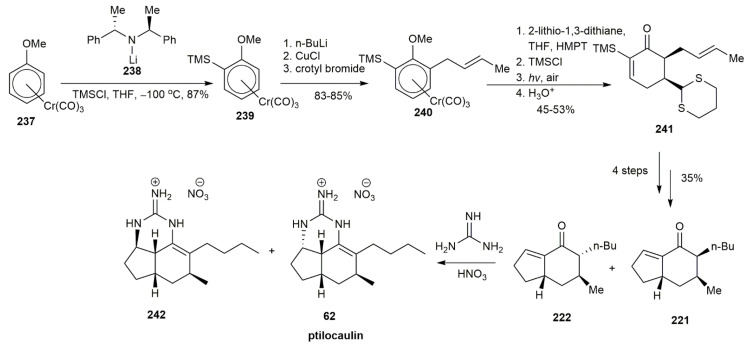
Schellhaas, synthetic approach to (+)-ptilocaulin.

Using a new approach, Schellhaas and his co-workers used a chiral arene-Cr(CO)_3_ complex as a starting material, due to its reactivity and stereochemistry [56] (Figure 30). Additionally, this complex is favorable because of its stereodirecting and activating functionality. The anisole complex **237** underwent enantioselective *O*-silylation using the chiral base **238**. The product, **239**, was obtained with a good yield of 87%. The *O*-methylation of **239** yielded compound **240** with an unsaturated side chain at the ortho position to the methoxide. Nucleophilic addition triggers a tele-substitution of **240.** Hence, the acid-free TMSCl and HMPA were added to inhibit the tele-substitution. After light-induced decomplexation and acid hydrolysis, the desired enone **241** was obtained in a yield ranging from 45 to 53%. The enone **241** was then subjected to conjugate addition using a Grignard reagent of 2-bromoethyl-1,3-dioxolane, followed by aldol cyclization, affording the hydroindenone **221** and **222** in a 1:1 ratio. The hydroindenone mixture was subjected to reflux in the presence of guanidine, followed by protonation with diluted nitric acid, to afford ptilocaulin **62** and its C-3a-diastereomer **242**.

**Figure 31:**
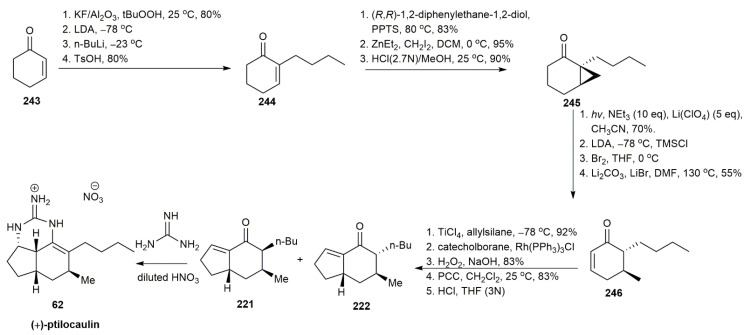
Cossy, synthetic approach to (+)-ptilocaulin.

Through the former study by Cossy and Furet, it was demonstrated that 3-methylcycloalkanones can be synthesized via the photoreduction of alkyl-substituted bicyclo [4.1.0] heptanones [62] (Figure 31). Cossy and Bouzbouz utilized the same strategy to synthesize (+)-ptilocaulin [60]. Cyclohexenone **243** first underwent epoxidation followed by the addition of a butyl group at the *α*-position and acid workup to yield compound **244** in an 80% yield. To form the bicyclo [4.1.0] heptanone structure, **244** was reacted with (*R*,*R*)-1,2-diphenylethane-1,2-diol in the presence of PPTS to form an acetal. The acetal was subjected to Simmons–Smith cyclopropanation and hydrolysis to afford **245**. The irradiation of **245** in the presence of triethylamine and LiCO_3_ afforded a ketone, which underwent a sequential bromination and debromination to afford a 55% yield of the enone **246**. The hydroboration of the alkene using Rh(PPh)_3_Cl, followed by oxidation using H_2_O_2_, yielded an aldehyde which, upon subsequent oxidation using PCC and acid hydrolysis, generated bicyclic enones **221** and **222** in a 65% yield. The addition of guanidine formed the final target compound of (+)-ptilocaulin **62**. 

**Figure 32:**
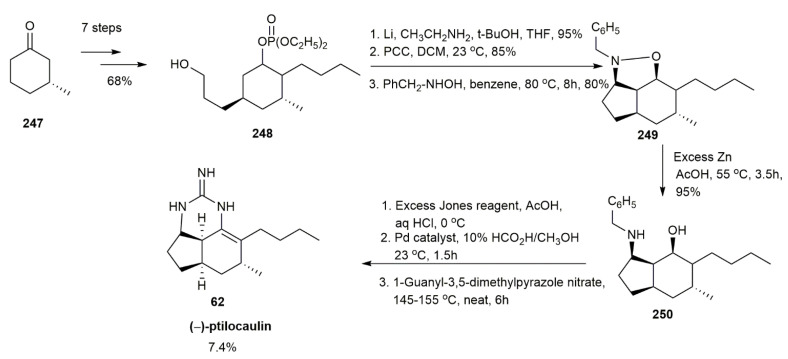
Roush, synthetic approach to (−)-ptilocaulin.

An approach utilizing intramolecular nitrone cyclization for synthesizing (−)-ptilocaulin was implemented by Roush and Walts [58] (Figure 32). The starting material, (*R*)-(+)-3-methylcyclohexanone **247**, underwent sulfenylation, oxidation, and selective hydroboration to obtain **248** in a 68% yield. The subsequent treatment with excess lithium in ethylamine yielded alcohol, which was then oxidized with PCC to afford an aldehyde. This aldehyde was then converted to isoxazolidine **249** through a nitrone intermediate. The isoxazolidine’s nitrogen-oxygen bond was cleaved by zinc in acetic acid to yield compound **250** in a 95% yield. A further reaction with Jones reagent oxidized **250**, followed by deprotection and condensation with guanidine to afford the (−)-ptilocaulin **62**. 

#### 3.2.2. 7-Epineoptilocaulin and Mirabilin B

**Figure 33:**
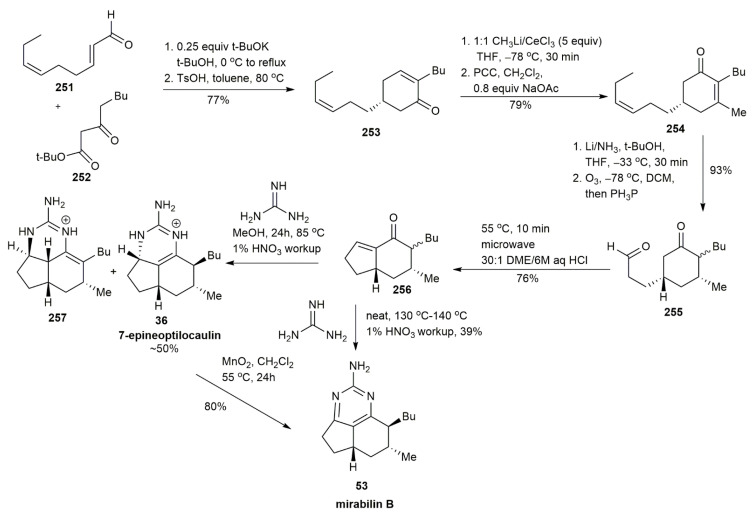
Yu, synthetic approach to 7-epineoptilocaulin and mirabilin B.

In 2009, the first attempt to synthesize other tricyclic guanidine compounds was conducted by Yu et al., upon the discovery of the disproportionation reaction of ptilocaulin by Murthy and Hassner [40,57] (Figure 33). The Robinson annulation of **251** to **252** afforded cyclohexenone **253** in a 77% yield. The presence of excess MeLi and CeCl3 afforded the conversion of cyclohexenone **253** into alcohol, followed by PCC-mediated oxidation to yield cyclohexenone **254** in a 79% yield. The successive Birch reduction of **254** afforded a 10:1 mixture of stereoisomers. The subsequent ozonolysis followed by triphenylphosphine reduction generated compound **255** in a 93% yield. To obtain the enone **256**, an intramolecular aldol condensation reaction was conducted using microwave irradiation. The enone was then refluxed with guanidine to afford 7-epineoptilocaulin **36** and **257**. It was also observed that heating the enone with guanidine in methanol for 24 h, followed by nitric acid workup, gave a better yield of around 50% 7-epineoptilocaulin **36** and 10% 3a,7-bisepiptilocaulin **257**. Two routes were used to synthesize mirabilin B **53**. In the first method, the oxidation of 7-epineoptilocaulin **36** with activated MnO_2_ afforded mirabilin B **53** in an 80% yield. The second method involved heating the bicyclic enone **256** at a higher temperature (130 ℃ to 140 ℃) for 4 h, followed by a nitric acid workup. The latter approach afforded mirabilin B **53** with a significantly lesser yield of 39%.

#### 3.2.3. Netamine E and Netamine G

**Figure 34:**
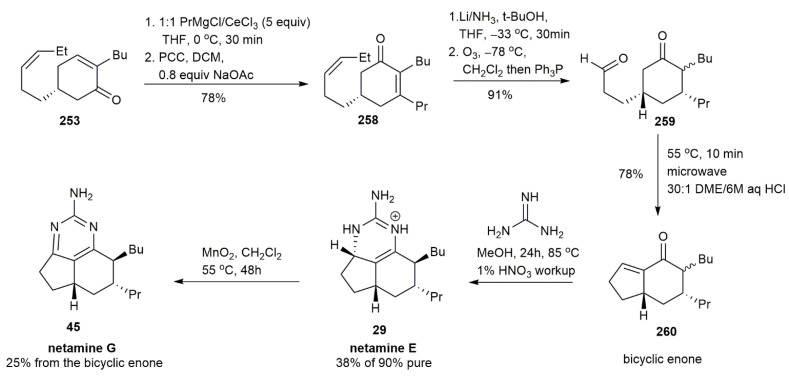
Yu, synthetic approach to netamine E and netamine G.

Yu et al. synthesized netamine E **29** and netamine G **45** using the same materials, **251** and **252**, that were employed for preparing 7-epineoptilocaulin **36** and mirabilin B **53** [40] (Figure 34). Both the starting materials, **251** and **252**, in the presence of a 10 mol % catalyst and *p*-toluenesulfonic acid, generated the enone **253***,* which, after subsequent steps, yielded netamine E **29** with a propyl side chain instead of methyl, as observed in 7-epineoptilocaulin **36** and mirabilin B **53**. The oxidation of netamine E **29** afforded netamine G **45** from the bicyclic enone, with a 25% yield. 

#### 3.2.4. Netamine C

**Figure 35:**
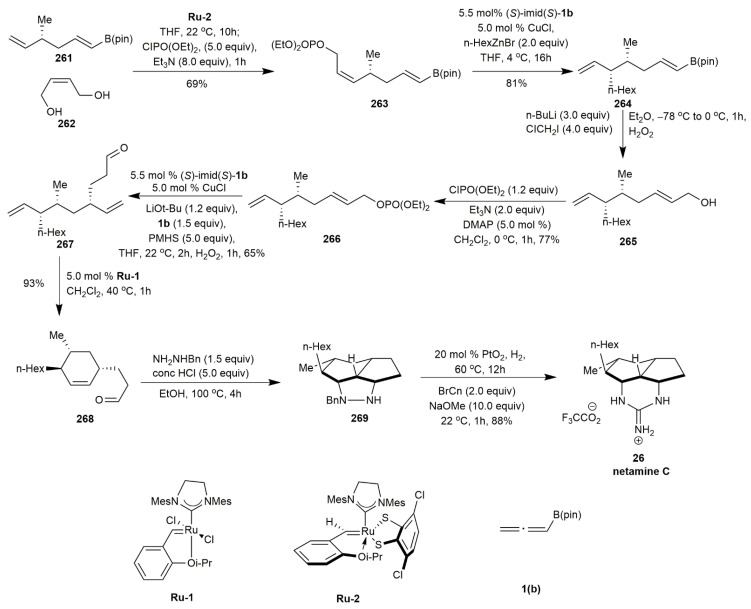
Sun, synthetic approach to netamine C.

Netamine C **26** is the latest compound to be synthesized from this tricyclic guanidine skeleton. Sun et al. employed copper-hydride-catalyzed allenylboronate synthesis to prepare netamine C **26** [39] (Figure 35). The cross-metathesis of **261** and **262** in the presence of **Ru-2** as a catalyst yielded **263** in a 69% yield. The product was then subjected to catalytic S_N_2 reaction and allylic substitutions in the presence of alkyl zinc halide reagent and (*S*)-imid(*S*)-**1b** to yield **264**. This was the first study on the reactivity of alkyl zinc halide. The next approach was to change the side chain of alkenyl-B (pin). The side chain of **264** was transformed into allylic alcohol **265** and then into phosphate **266**. The gamma-alkyl substituted aldehyde **267** was obtained through another allylic substitution with **1b** in the presence of (*S*)-imid(*S*)-**1b**. Further ring-closing metathesis of **267** afforded the cyclohexenyl intermediate **268** in a 93% yield. The subsequent [3+2] cycloaddition with *N*-benzyl hydrazone provided **269**, which was converted into netamine C **26** in an 88% yield. 

## 4. Conclusions

The compounds comprising fused tricyclic guanidine moieties have been reported to exhibit a wide range of bioactivities, such as anti-cancer, antiviral, antimicrobial, and anti-malarial properties. Two main skeletons of tricyclic guanidine are exhibited by batzelladine and ptilocaulin. Netamines and mirabilins represent other derivations of ptilocaulins that exhibit significant bioactivities by inhibiting cancer cell lines. Various synthetic strategies have been employed to approach these skeletons over the years. In particular, the asymmetric version of the total synthesis of batzelladine C and F somewhat resolved the stereochemical ambiguities. Several synthetic approaches to the synthesis of ptilocaulins have been reported, with most of the strategies targeting the same precursor, the Snider ketone, which can ultimately be converted into ptilocaulin. On the contrary, only two studies have reported the synthesis of mirabilin and netamine, despite their potent bioactivities. Thus, there is still room for research aiming to achieve the total synthesis of these bioactive fused tricyclic guanidines for the further investigation of their biological potential. In addition, there is a need to create new approaches to the synthesis of these skeletons more economically and efficiently, especially in regard to the synthesis of less widely reported mirabilins and netamines, in order to pave the way for the further exploration of their biological potential and their plausible development as therapeutic drugs. 

## Figures and Tables

**Table 1:** Bioactivities of batzelladines.

	Sponge Source	Synthesized	Anti-Cancer	Anti-Malarial	Anti- Microbial	HIV Inhibitor	Reference
**Skeleton 1**							
Batzelladine A (**1**)	*Batzella* sp., *Monanchora arbusculla*, *Clathria calla*	/		/		/	[1,2,3,4]
Norbatzelladine A (**2**)	*M. arbuscula*, *C. calla*		/	/			[2]
Dinorbatzelladine A (**3**)	*M. arbuscula*, *C. calla*		/	/			[2]
Batzelladine D (**4**)	*Batzella* sp., *M. arbuscula*	/			/	/	[1,3,5,6,7,8,9]
Batzelladine F (**5**)	*Batzella* sp., *M. arbuscula*	/			/	/	[5,10,11,12,13,14]
Batzelladine G (**6**)	*Batzella* sp.					/	[10]
Batzelladine K (**7**)	*M. unguifera*	/					[15,16]
Batzelladine L (**8**)	*M. unguifera*, *M. arbuscula*, *C. calla*		/	/	/	/	[2,5,16]
Norbatzelladine L (**9**)	*M. arbuscula*, *C. calla*		/	/	/		[2,5]
Merobatzelladine A (**10**)	*Monanchora* sp.			/	/		[17]
Merobatzelladine B (**11**)	*Monanchora* sp.	/		/	/		[17,20,21]
**Skeleton 2**							
Batzelladine B (**12**)	*Batzella* sp.	/				/	[1,22,23]
Batzelladine E (**13**)	*Batzella* sp.	/					[1,24]
**Skeleton 3**							
Batzelladine C (**14**)	*Batzella* sp., *M. unguifera*	/	/	/	/	/	[1,16,25]
Batzelladine J (**15**)	*M. unguifera*		/				[26]
Batzelladine M (**16**)	*M. unguifera*		/	/	/	/	[16]
Batzelladine N (**17**)	*M. unguifera*		/		/	/	[16]
**Skeleton 4**							
Batzelladine H (**18**)	*Batzella* sp.					/	[10]
Batzelladine I (**19**)	*Batzella* sp.					/	[10]
Dinordehydrobatzelladine B (**20**)	*M. arbuscula*, *C. calla*		/	/			[2]
Dihomodehydrobatzelladine C (**21**)	*M. arbuscula*, *C. calla*		/	/			[2]
Clathriadic acid (**22**)	*M. arbuscula*, *C. calla*		/	/			[2]
**Skeleton 5**							
Dehydrobatzelladine C (**23**)	*M. unguifera*		/	/	/	/	[16]

/ = exhibited bioactivities.

**Table 2:** Bioactivities of ptilocaulin, netamine, and mirabilin.

	Sponge Source	Synthesized	Anti-Cancer	Anti-Malarial	Antimicrobial	Hemolytic Activities	Reference
**Saturated**							
Netamine A (**24**)	*Biemna laboutei*						[34]
Netamine B (**25**)	*Biemna laboutei*						[34]
Netamine C (**26**)	*Biemna laboutei*	/	/				[34,39]
Netamine D (**27**)	*Biemna laboutei*		/				[34]
Mirabilin K (**28**)	*Acanthella cavernosa*						[37]
**Δ ^11,12^**							
Netamine E (**29**)	*Biemna laboutei*	/					[34,40]
Netamine O (**30**)	*Biemna laboutei*		/	/			[36]
Mirabilin H (**31**)	*Clathria* sp.		/				[33]
Netamine P (**32**)	*Biemna laboutei*			/			[36]
Netamine Q (**33**)	*Biemna laboutei*		/	/			[36]
Netamine R (**34**)	*Biemna laboutei*						[36]
Netamine S (**35**)	*Biemna laboutei*						[36]
7-Epineoptilocaulin (**36**)	*Batzella* sp.	/					[29,40]
Mirabilin E (**37**)	*Arenochalina mirabilis*						[31]
8α-Hydroxy-7-epineoptilocaulin (**38**)	*Batzella* sp.						[29]
Mirabilin E diacetate (**39**)	*Arenochalina mirabilis*						[31]
Mirabilin D (**40**)	*Arenochalina mirabilis*						[31]
Mirabilin J (**41**)	*Clathria* sp.		/				[33]
Mirabilin D diacetate (**42**)	*Arenochalina mirabilis*						[31]
**Δ ^9,10^**							
Isoptilocaulin (**43**)	*Ptilocaulis* aff. *Ptilocaulis spiculifer*		/		/		[27]
**Pyrimidines**							
Netamine F (**44**)	*Biemna laboutei*						[34]
Netamine G (**45**)	*Biemna laboutei*	/					[34,40]
Netamine H (**46**)	*Biemna laboutei*						[35]
Netamine I (**47**)	*Biemna laboutei*						[35]
Netamine J (**48**)	*Biemna laboutei*						[35]
Mirabilin A (**49**)	*Arenochalina mirabilis, Biemna laboutei*			/			[31,35]
Mirabilin C (**50**)	*Arenochalina mirabilis, Biemna laboutei, Clathria* sp.		/				[31,33,35]
Mirabilin A acetate (**51**)	*Arenochalina mirabilis*						[31]
Mirabilin C acetate (**52**)	*Arenochalina mirabilis*						[31]
Mirabilin B (**53**)	*Arenochalina mirabilis, Monanchora unguifera*	/	X		/		[30,31,41]
Mirabilin B acetate (**54**)	*Arenochalina mirabilis*						[31]
8*α*-Hydroxymirabilin (**55**)	*Batzella* sp., *Monanchora unguifera*						[29,30]
8*β*-Hydroxymirabilin (**56**)	*Monanchora unguifera*						[30]
**Δ ^10,11^**							
Netamine K (**57**)	*Biemna laboutei*			/			[35]
Netamine L (**58**)	*Biemna laboutei*						[35]
Netamine M (**59**)	*Biemna laboutei*		/				[35,37]
Mirabilin G (**60**)	*Clathria* sp.		/		/		[32,33,37]
Netamine N (**61**)	*Biemna laboutei*						[35]
Ptilocaulin (**62**)	*Ptilocaulis* aff., *Ptilocaulis spiculifer*	/	/		/	/	[27,41,42]
8*β*-Hydroxyptilocaulin (**63**)	*Monanchora arbuscula*		/			/	[28,41]
Mirabilin F (**64**)	*Arenochalina mirabilis*		/				[31,33]
Mirabilin F acetate (**65**)	*Arenochalina mirabilis*						[31]
Mirabilin I (**66**)	*Clathria* sp.		/				[33]

/= exhibit bioactivity, X = did not exhibit any bioactivity.

**Table 3:** Specification of the bioactivities of batzelladines, ptilocaulin, netamines, and mirabilins.

Common Names	Mode of Action/Cells Inhibited	References
**HIV inhibitor**		
Batzelladine A (**1**)	Inhibits gp120 binding to CD4, protein kinase C activity, binding of interleukin-8 (IL8) and calcitonin gene-related peptide (CGRP) to their receptors, inhibits Vero cells	[1]
Batzelladine D (**4**)	Vero cells	[1]
Batzelladine F (**5**)	Induces p56^lck^-CD4 dissociation	[10]
Batzelladine G (**6**)		
Batzelladine L (**8**)	Shows inhibitory activity against human HIV-1 virus	[16]
Batzelladine B (**12**)	Inhibits gp120 binding to CD4, protein kinase C activity, binding of interleukin-8 (IL8) and calcitonin gene-related peptide (CGRP) to their receptors, inhibits Vero cells	[1]
Batzelladine C (**14**)	Vero cells, shows inhibitory activity against the human HIV-1 virus	[1,16]
Batzelladine M (**16**)	Shows inhibitory activity against human HIV-1 virus	[16]
Batzelladine N (**17**)		
Batzelladine H (**18**)	Induces p56^lck^-CD4 dissociation when combined with batzelladine I	[10]
Batzelladine I (**19**)	Induces p56^lck^-CD4 dissociation when combined with batzelladine H	
Dehydrobatzelladine C (**23**)	Shows inhibitory activity against human HIV-1 virus	[16]
**SARS-CoV-2 inhibitor**		
Batzelladine H (**18**)	Inhibits SARS-CoV-2 main protease (M^pro^)	
Batzelladine I (**19**)		
Mirabilin G (**60**)	Inhibits SARS-CoV-2 main protease (M^pro^)	
**Anti-cancer**		
Norbatzelladine A (**2**)	MDA-MB-231, A549, HT29	[2]
Dinorbatzelladine A (**3**)		
Batzelladine L (**8**)	DU-145, IGROV, SK-BR3, leukemia L-562, PANCL, HeLa, SK-MEL-28, A549, HT-29, LOVO, and LOVO-DOX	[16]
Norbatzelladine L (**9**)	MDA-MB-231, A549, HT29	[2]
Batzelladine C (**14**)	DU-145, IGROV, SK-BR3, leukemia L-562, PANCL, HeLa, SK-MEL-28, A549, HT-29, LOVO, and LOVO-DOX	[16]
Batzelladine J (**15**)	P-388, A-549, HT-29, MEL-28, DU-145	[26]
Batzelladine M (**16**)	DU-145, IGROV, SK-BR3, leukemia L-562, PANCL, HeLa, SK-MEL-28, A549, HT-29, LOVO, and LOVO-DOX	[16]
Batzelladine N (**17**)		
Dinordehydrobatzelladine B (**20**)	A549, HT29	[2]
Dihomodehydrobatzelladine C (**21**)	MDA-MB-231, A549, HT29	
Clathriadic acid (**22**)		
Dehydrobatzelladine C (**23**)	DU-145, IGROV, SK-BR3, leukemia L-562, PANCL, HeLa, SK-MEL-28, A549, HT-29, LOVO, and LOVO-DOX	[16]
Netamine C (**26**)	A549, HT29, MDA-MB-231	[34]
Netamine D (**27**)		
Netamine O (**30**)	KB tumor cell	[36]
Mirabilin H (**31**)	SH-SY5Y, AGS, HT29, Intestine-407	[33]
Netamine Q (**33**)	KB tumor cells	[36]
Mirabilin J (**41**)	SH-SY5Y, AGS, HT29, Intestine-407	[33]
Isoptilocaulin (**43**)	L1210 leukemia cells	[27]
Mirabilin C (**50**)	SH-SY5Y, AGS, HT29, Intestine-407	[33]
Netamine M (**59**)	KB cell, HEK 293 cells. Inhibits TPA-induced degradation of PDCD4	[35,37]
Mirabilin G (**60**)	HEK 293, SH-SY5Y, AGS, HT29, Intestine-407, stabilizes TPA-induced degradation of PDCD4	[33,37]
Ptilocaulin (**62**)	L1210, MCF-7, B16F10, HL-60, and MDA-MB-435	[27,41,42]
8b-Hydroxyptilocaulin (**63**)	HL-60 and MDA-MB-435	[41]
Mirabilin F (**64**)	SH-SY5Y, AGS, HT29, Intestine-407	[33]
Mirabilin I (**66**)		
**Anti-malarial**		
Batzelladine A (**1**)	*P. falciparum* (FcB1)	[2]
Norbatzelladine A (**2**)		
Dinorbatzelladine A (**3**)		
Batzelladine L (**8**)	*P. falciparum* (FcB1) and its D6 clone and W2 clone	[2,16]
Norbatzelladine L (**9**)	*P. falciparum* (FcB1)	[2]
Merobatzelladine A (**10**)	*P. falciparum*	[17]
Merobatzelladine B (**11**)		
Batzelladine C (**14**)	Against *P. falciparum* D6 clone and W2 clone	[16]
Batzelladine M (**16**)		
Dinordehydrobatzelladine B (**20**)	*P. falciparum* (FcB1)	[2]
Dihomodehydrobatzelladine C (**21**)		
Clathriadic acid (**22**)		
Dehydrobatzelladine C (**23**)	Against *P. falciparum* D6 clone and W2 clone	[16]
Netamine O (**30**)	*P. falciparum*	[36]
Netamine P (**32**)		
Netamine Q (**33**)		
Mirabilin A (**49**)		[35]
Netamine K (**57**)		
**Antimicrobial**		
Batzelladine D (**4**)	*Trypanosoma cruzi* trypomastigotes, *Leishmania infantum* promastigotes, *Saccharomyces cerevisiae*	[5,18]
Batzelladine F (**5**)	*Trypanosoma cruzi* trypomastigotes, *Leishmania infantum* promastigotes	[5]
Batzelladine L (**8**)	*T. cruzi* trypomastigotes, *L. infantum* promastigotes Strong activities against AIDS-OIs *Candida albicans*, *Cryptococcus neoformans*, *S. aureus*, methicillin-resistant *S. aureus* (MRS), *Pseudomonas aeruginosa*, and *M. intracellulare*, as well as *Aspergillus fumigatus*, *M. tuberculosis*, and *Leishmania donovani*	[5,16]
Norbatzelladine L (**9**)	*T. cruzi* trypomastigotes, *L. infantum* promastigotes, *S. cerevisiae*	[5]
Merobatzelladine A (**10**)	*Vibrio anguillarum*, *Trypanosoma brucei brucei*	[17]
Merobatzelladine B (**11**)		
Batzelladine C (**14**)	Strong activities against AIDS-OIs *C. albicans, C. neoformans*, *S. aureus*, methicillin-resistant *S. aureus* (MRS), *P. aeruginosa*, *M. intracellulare*, and *A. fumigatus*	[16]
Batzelladine M (**16**)		
Batzelladine N (**17**)	*M. tuberculosis*	[16]
Dehydrobatzelladine C (**23**)	Strong activities against AIDS-OIs *C. albicans*, *C. neoformans*, *S. aureus*, methicillin-resistant *S. aureus* (MRS), *P. aeruginosa*, *M. intracellulare*, and *A. fumigatus*	[16]
Isoptilocaulin (**43**)	*S. pyogenes*, *S. pneumoniae*, *E. faecalis*, *S. aureus*, *E. coli*	[27]
Mirabilin B (**53**)	*C. neoformans*, *L. donovani*	[30]
Mirabilin G (**60**)	*E. coli*, *Serratia marcescens,* and *S. cerevisiae*	[32]
Ptilocaulin (**62**)	*Streptococcus pyogenes*, *Streptococcus pneumoniae*, *E. faecalis*, *S. aureus*, *E. coli*	[27]
**Hemolytic activities**		
Ptilocaulin (**62**)	The plasma membrane of mouse erythrocytes	[41]
8*β*-Hydroxyptilocaulin (**63**)		

## Data Availability

Not applicable.
